# Optical Activity Modulation in Chiral Metasurfaces via Structured Light

**DOI:** 10.1021/acs.nanolett.5c03044

**Published:** 2025-08-05

**Authors:** Paula L. Lalaguna, Shun Hashiyada, Nikolaj Gadegaard, Jörg B. Götte, Stephen M. Barnett, Kayn A. Forbes, Yoshito Y. Tanaka, Malcolm Kadodwala

**Affiliations:** † School of Chemistry, 3526 University of Glasgow, Glasgow G12 8QQ, United Kingdom; ‡ Research Institute for Electronic Science, 12810 Hokkaido University, Sapporo, Hokkaido 001-0021, Japan; § School of Engineering, Rankine Building, Glasgow G12 8LT, United Kingdom; ∥ SUPA, School of Physics and Astronomy, University of Glasgow, Glasgow G12 8QQ, United Kingdom; ⊥ School of Chemistry, 6106 University of East Anglia, Norwich Research Park, Norwich NR4 7TJ, United Kingdom

**Keywords:** orbital angular momentum, vortex beams, chirality, optical activity, chiral metamaterials

## Abstract

We demonstrate a real-time, all-optical method for modulating optical activity in chiral metasurfaces using structured light without altering the metasurface geometry. By employing tightly focused Laguerre–Gaussian beams carrying spin and orbital angular momentum, we achieve dynamic control of the dichroism through selective excitation of optically dark multipolar modes. Unlike conventional methods reliant on thermal, mechanical or chemical stimuli, our approach offers noninvasive, and reversible modulation, overcoming key limitations in response time and energy efficiency. The structured light’s field gradients enable access to optical modes otherwise inactive under plane wave illumination. This technique opens new pathways for adaptive nanophotonic systems, including quantum sensors, polarization controllers, and quantum encryption platforms, where high-speed and contactless tuning of chiroptical properties is essential.

## Graphical abstract

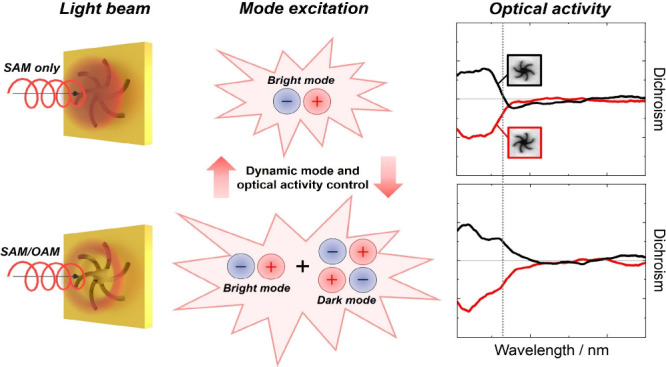

The control of optical activity in chiral metamaterials has progressed from static geometric design to dynamic reconfigurable systems modulated by external stimuli. Mechanisms such as nonlinear optical pumping,^1^ thermally induced phase transitions^2^ and electrically responsive designs^3,4^ have enabled active tuning of circular dichroism and optical rotation. Yet these strategies are typically constrained by limited modulation speeds, energy in efficiencies and challenges in scalability due to complexity.

All-optical control offers a compelling alternative. However, most reported methods rely on high-power pulsed lasers operating in nonlinear regimes,^5^ posing integration and thermal challenges. Continuous-wave (CW) approaches, including photothermal or phase-change actuation,^6,7^ offer improved energy compatibility but remain limited by slow response speeds and dependence on often irreversible material changes.

Here, we present a structured-light-based approach that addresses these limitations. Unlike previous studies where orbital angular momentum (OAM) was treated as an alternative to circularly polarized light,^8–12^ our findings show that OAM’s function is not to directly induce optical activity. Rather, under tight focusing, the beam’s spatial field gradients—enabled by SAM-OAM beam structure—drive excitation of dark multipolar modes. This reframes OAM not as a chiral agent but as a field-engineering mechanism for dynamic control of optical activity.

The metasurfaces used in this study have been described in detail elsewhere^13^ and they comprise periodic arrays of shuriken-shaped nanoindentations with 6-fold symmetry. The nanoindentations are arranged in a square lattice with a periodicity of 720 nm. Each indentation is 80 nm deep and is conformally coated with a 100 nm layer of gold to produce a continuous, planar chiral metallic film (Figure 1). The in-plane asymmetry of the shuriken motifs results in broken mirror symmetry, giving rise to strong optical activity.^13^

**Figure 1:**
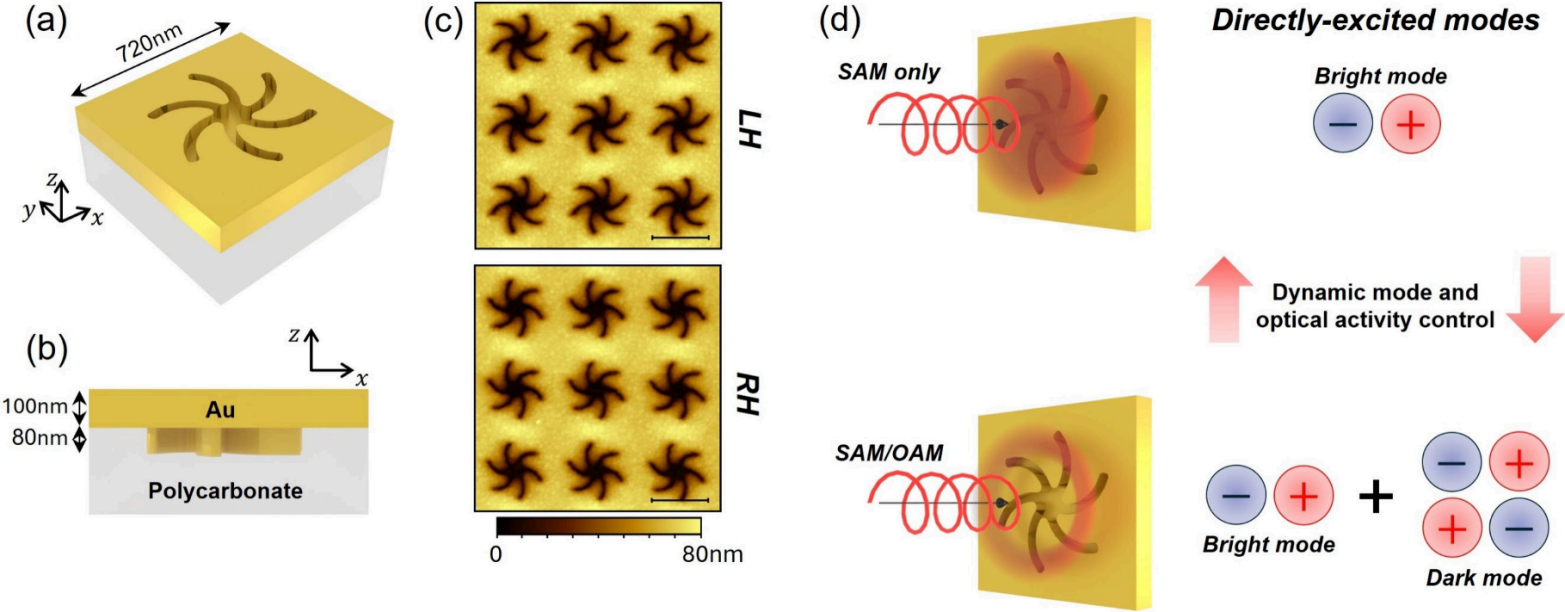
Chiral gold metasurfaces used in this work. (a) Schematic of a chiral shuriken-shaped nanoindentation; the dimensions of a single unit cell are shown. (b) Side view of the unit cell in (a). (c) AFM images of the left-handed (LH) and right-handed (RH) nanoindentations (scale bar: 500 nm). (d) An illustration of the concept of this work, showing that modal excitation and optical activity of nanostructures can be tuned dynamically by adding or removing OAM to the SAM of a tightly focused circularly polarized beam.

Previous measurements in water have shown that the optical activity of the shuriken-shaped metasurfaces arises from spin-dependent near-field coupling between bright and dark plasmonic modes.^14^ This coupling produces a plasmon-induced reflectance (PIR) minimum in the reflectance spectra, the presence and spectral shape of which depend on the handedness of the incident circularly polarized light. When the spin angular momentum (SAM) of the incident light matches the handedness of the metasurface, strong near-field coupling between an electric dipole mode (bright) and an electric quadrupole mode (dark) leads to the formation of a pronounced PIR feature. When the handedness is mismatched, this coupling is suppressed and the PIR feature diminishes, demonstrating the chiral selectivity of the optical response. This helicity-dependent mechanism is described in detail by Kelly et al.^14^

To investigate how this response is modified under structured light illumination and in a lower-index environment, measurements were collected under weakly focused beam conditions (numerical aperture NA = 0.3) in air. Preliminary characterization with a Gaussian beam was used in conjunction with Stokes polarimetry to measure the optical rotatory dispersion (ORD) and extinction spectra of the metasurfaces. ORD was obtained by analyzing the change in polarization of reflected linearly polarized light, and the corresponding circular dichroism (CD) spectrum was derived via Kramers–Kronig transformation. The ORD and CD spectra (Figure S1) exhibited opposite signs for the two enantiomeric forms of the metasurface and were qualitatively similar to those previously measured in water. However, both the spectral position of the chiroptical features and the plasmonic resonance observed in reflectance were blue-shifted in air, consistent with the lower refractive index of the surrounding medium. In addition, the PIR dip was smaller in air compared to water, indicative of a slightly weaker level of coupling (Figure S1).

Structured light measurements were performed using a helical dichroism spectrometer to probe the response to structured light.^200^ Three beam configurations were tested: (i) a circularly polarized Gaussian beam carrying SAM only (σ = ± 1), corresponding to conventional CD; (ii) a LG beam with topological charge *l* = ± 1 and linear polarization (σ = 0), carrying OAM only; and (iii) circularly polarized LG beams with *l* = ± 1 and antiparallel SAM-OAM combinations (σ = ± 1, *l* = ∓1). Dichroic spectra were acquired at three randomly selected positions on the metasurface, with no significant variation observed (Figure S4), confirming spatial uniformity of the optical response.

The dichroic spectra measured using the SAM only and SAM-OAM (antiparallel) configurations were identical (Figure 2) and matched the CD spectrum derived from Kramers–Kronig transformation of the optical ORD measured using Stokes polarimetry. In contrast, the OAM-only configuration produced no measurable dichroism (Figure S4). These results demonstrate that, under weak focusing, the chiroptical response of the metasurface arises exclusively from spin angular momentum. The presence of orbital angular momentum, at least for beams with |*l*| = 1, does not influence the optical activity in either isolated or combined form when SAM and OAM are antiparallel. This observation is consistent with prior findings that chiral microstructures do not exhibit appreciable helical dichroism unless the OAM topological charge is sufficiently high (e.g., |*l*| ≥ 4), such that the helical phase structure of the beam effectively overlaps with the chiral geometry of the target.^15^ For our nanostructure with a size of ∼ 500 nm there is small spatial overlap between the *l* = 1 beam under weak focusing.

**Figure 2:**
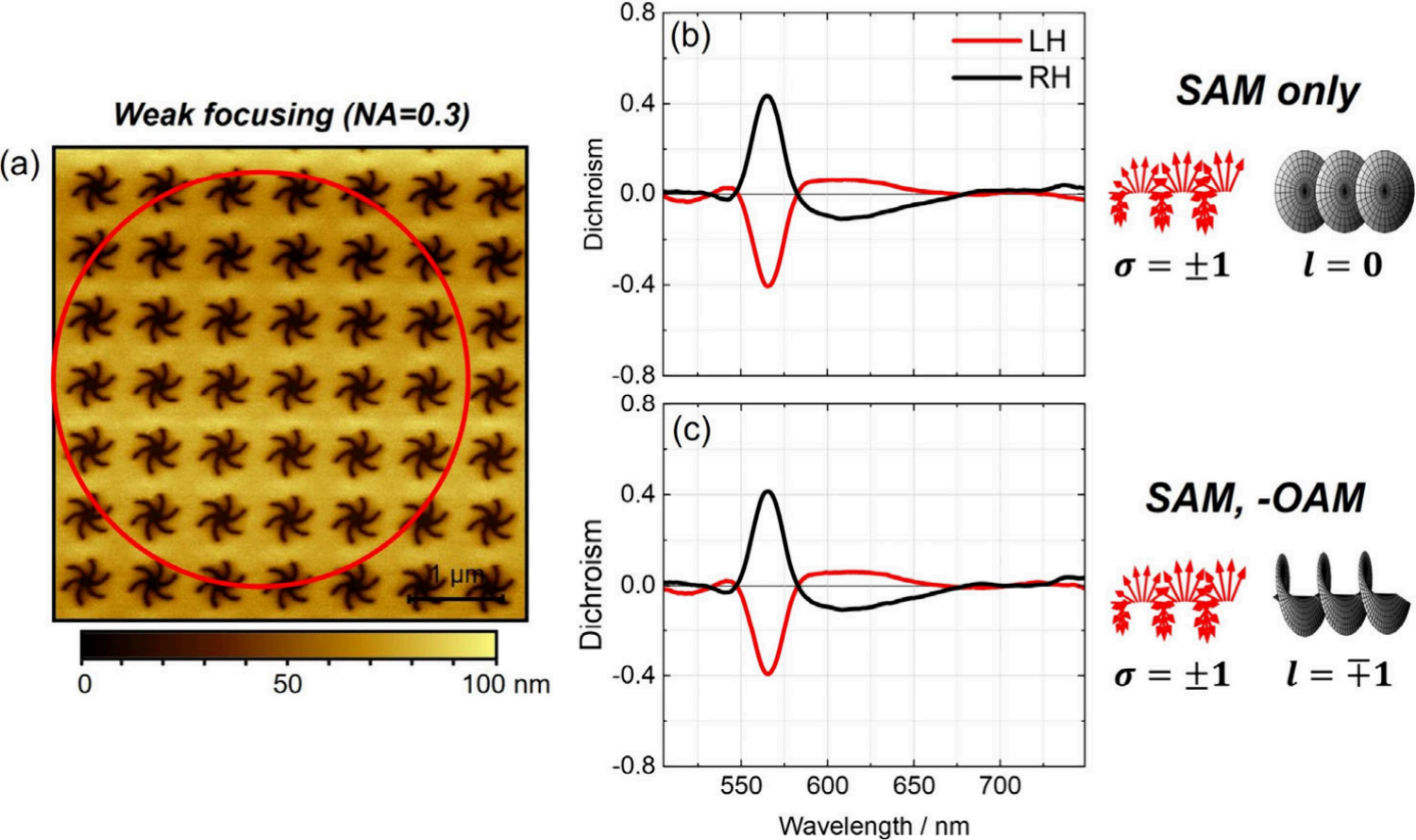
Experimental data for weakly focused (NA = 0.3) beams. (a) AFM image of the periodic shuriken nanoindentations with the approximate beam diameter of the Gaussian beam (defined as 2*w*_0_) indicated as a red circle (scale bar: 1 μm). The beam spot size covers approximately 6 lattice spacings. (b, c) Dichroism data for (b) SAM only and (c) antiparallel SAM-OAM combination.

This behavior aligns with the structure of paraxial LG beams, in which the electric field gradient responsible for OAM-matter coupling is given by

1 $$\nabla E_{\mathrm{LG}}^{l,p=0}=\left[\mathrm{\rho ̂}\left(\frac{\left|l\right|}{\rho }-\frac{2\rho }{w_{0}^{2}}\right)+\mathrm{\phi ̂}\frac{il}{\rho }+ikẑ\right]E_{\mathrm{LG}}^{l,p=0}$$

As seen from eq 1, the electric field gradient scales as |*l*|/ρ, where ρ is the radial distance from the beam axis. These gradients decay rapidly with increasing ρ, and since most nanostructures lie outside the central high-gradient region, the OAM has no effect. Consequently, in the weak focusing regime, the metasurface exhibits a purely spin-driven optical response. This is verified with the numerical simulations performed under weak focusing, which show that the structures off the beam center show a dipole-driven dichroism, as opposed to structures at the beam center which show dark-mode excitations (Figures S14–S16). Even if the structure placed at the beam center shows dichroism due to higher-order modes, most of the structures, which are off the beam center, show the spin-driven optical activity.

To investigate the influence of beam confinement at the single-structure level, measurements were performed under tight focusing using a high numerical aperture (NA = 0.95) objective. At a wavelength of 600 nm, the beam waist was reduced to *w*_0_ = 647 ± 5 nm, closely matching the lateral dimensions of a single shuriken nanostructure. This enabled spatially resolved measurements of the chiroptical response at both ensemble and individual-structure levels.^16^

Dichroism data for the tightly focused regime is shown in Figure 3 and Figure 4. Three beam configurations were used, consistent with earlier weak focusing experiments: (i) SAM only (circularly polarized Gaussian beam), (ii) OAM only (linearly polarized LG beam with *l* = ± 1) and (iii) antiparallel SAM–OAM combinations (σ = ± 1 and *l* = ∓1). To obtain spatially averaged spectra, two complementary approaches were employed. In the first, full spectra were recorded at three randomly selected locations on the metasurface and averaged. These spectra exhibited pronounced spatial heterogeneity, with significant variation from point to point (Figure S5). In the second approach, a 2D dichroism map was recorded at a fixed wavelength by raster-scanning a 6.6 × 6.6 μm^2^ area using a piezo-controlled stage (Figure 3a). A circular mask with a diameter corresponding to a multiple of the lattice periodicity (720 nm) was applied to extract an area-integrated dichroic value. This method yielded a more representative spatial average.

**Figure 3:**
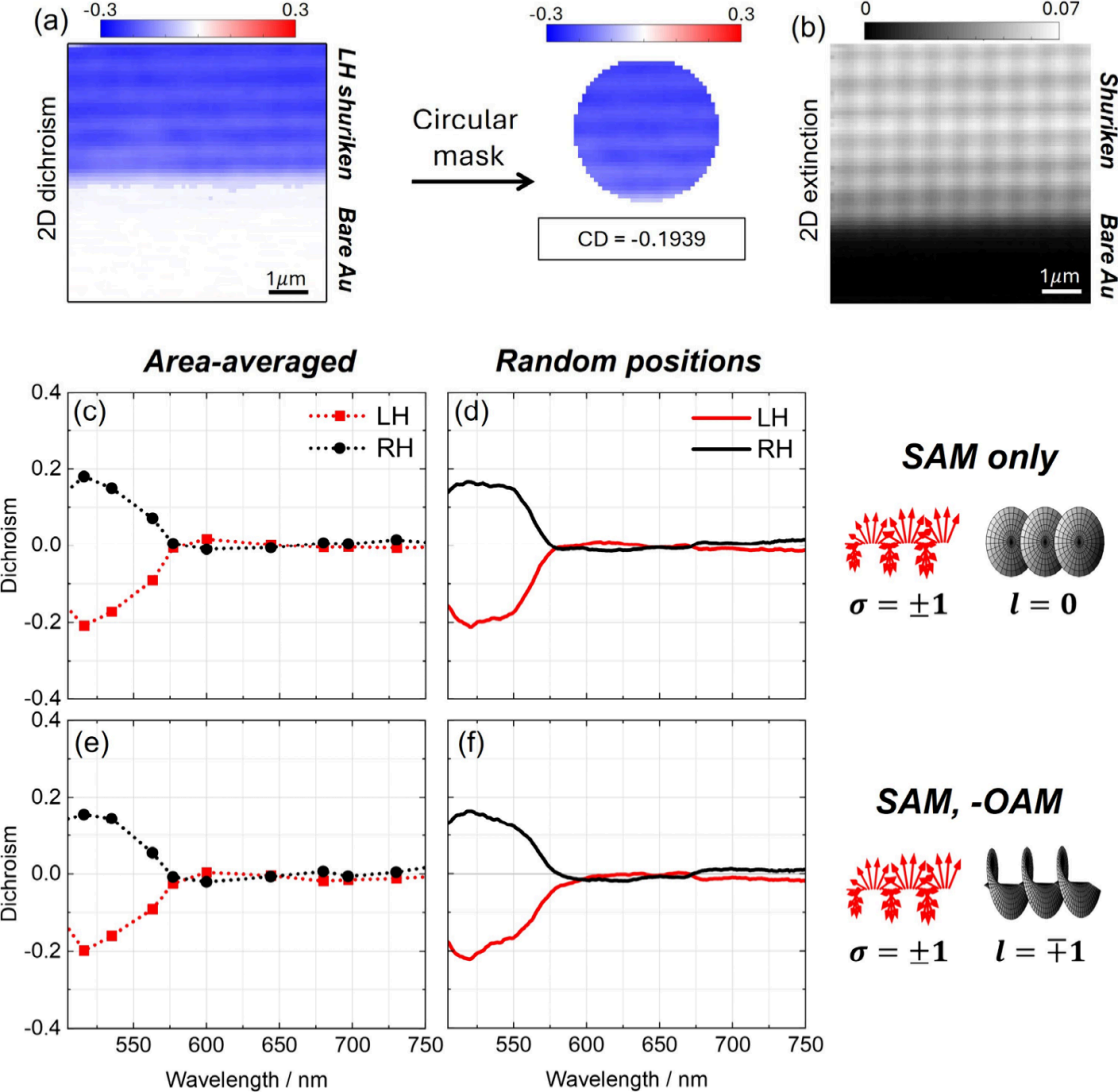
Experimental data dichroism for tightly focused (NA = 0.95) beams and spatial averaging. (a, b) Examples of 2D (a) dichroism and (b) extinction maps collected with *l* = 0 beam at λ = 535 nm in the LH shuriken structure. In both cases, the pixel resolution is 100 nm, and the scale bar is 1 μm. In (a), a circular mask can be applied to obtained an area-averaged dichroism value. The circle diameter is a multiple of the periodicity of the structure. (c, d) Dichroism for SAM only for (c) area-averaging using the circular mask in (a) and (d) average of 3 random sample positions. (e, f) Same dichroism as (c, d), respectively, for antiparallel SAM-OAM.

**Figure 4:**
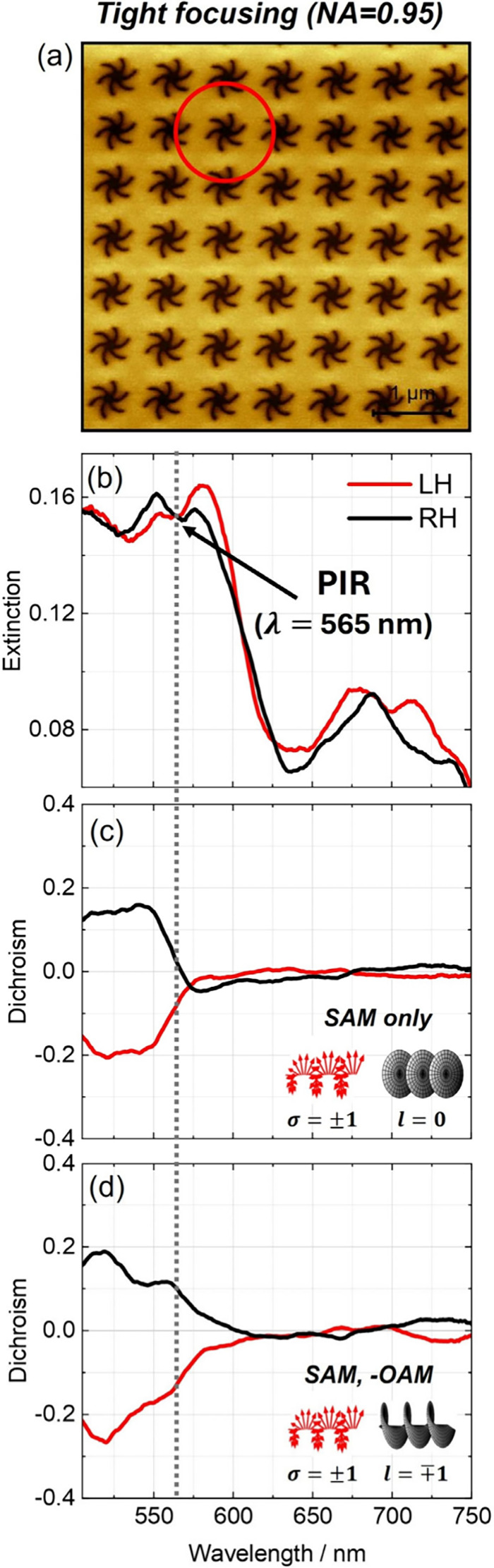
Experimental dichroism data for tightly focused (NA = 0.95) beams on a single structure. (a) AFM image of the periodic shuriken nanoindentations with the approximate beam diameter of the Gaussian beam (defined as 2*w*_0_) indicated as a red circle (scale bar: 1 μm). The beam spot size covers approximately slightly more than one lattice spacing. (b) Extinction data collected with the weakly focused beam showing PIR due to near-field coupling between bright and dark modes. (c, d) Dichroism data for (c) SAM only and (d) antiparallel SAM-OAM combination.

In both spatial averaging methods, no significant dichroic response was detected for OAM-only excitation (Figure S5). Furthermore, the SAM-only and SAM–OAM spectra were nearly identical in shape and amplitude (Figure 3c-f). Importantly, none of the tightly focused spectra resembled the dichroism spectra under weak focusing, nor did the extinction spectra display the PIR dip (Figure S6). This breakdown in spectral structure confirms that the coupled oscillator model – previously used to describe weak focusing results^14^ – is no longer valid. Since the beam interrogates only a single unit cell, coupling between bright (dipolar) and dark (nondipolar) modes that underpins the PIR is absent.^17^ This interpretation is further supported by plane-wave simulations of single, nonperiodic nanostructures, which show the absence of near-field coupling once the periodicity is removed (Figure S9–S10).^18^ Although tightly focused beams within a periodic structure could not be simulated directly, these single-structure calculations revealed the disappearance of the PIR feature when interstructure coupling was removed, consistent with the breakdown of lattice-mediated near-field interactions. Additionally, under tight focusing, the dichroism signal is notably blue-shifted below 550 nm and dominates the blue region of the spectrum, rather than exhibiting the typical plasmonic CD signature near 600 nm. This pronounced shift is attributed to strong interband transitions in gold, wherein electrons are excited from the filled d-band to the conduction band creating transient holes that couple asymmetrically to circularly polarized light and enhance chiral optical effects. Under such intense excitation, the modified hot-carrier distribution further amplifies these interband contributions, reshaping the CD line shape in the blue and suppressing the plasmonic feature near 600 nm, underscoring the importance of flux-dependent interband-mediated hot-electron dynamics in our measurements.^19,20^

To assess whether structured light could still modulate the chiroptical response at the single-object level, measurements were performed on individual nanostructures. Beam alignment was guided by two-dimensional extinction mapping, acquired by scanning the sample with a tightly focused *l* = 0 beam, shown in Figure 3b. The centers of the shuriken motifs appeared as distinct maxima due to enhanced extinction relative to the surrounding flat film.^14^ These features served as reliable markers to locate individual structures, enabling 100 nm positioning accuracy via a piezo-controlled stage.

At the center of the nanostructures, a significant enhancement in dichroism was observed at ∼ 565 nm under SAM-OAM excitation, whereas the SAM-only beam produced a much weaker signal at the same wavelength (Figure 4). This enhancement was reproducible and reversible (Figure S7), and could be toggled by switching the *q*-plate on or off. Notably, the enhancement occurred at a wavelength corresponding to the PIR dip observed under weak focusing, suggesting excitation of the dark mode.

We attribute this excitation not to the presence of orbital angular momentum itself, but to the field structure of the SAM-OAM beam. The superior transverse and longitudinal field gradients in LG beams compared to Gaussian beams enables direct excitation of higher-order (e.g., quadrupolar) modes. This is manifested in the electric field intensity on top of the nanostructure, which shows nondipolar (dark) excitations in the case of LG beams (Figure 5). This effect is not significant in the weakly focused regime due to the larger beam waist of the LG beam, resulting in smaller field gradients.

**Figure 5:**
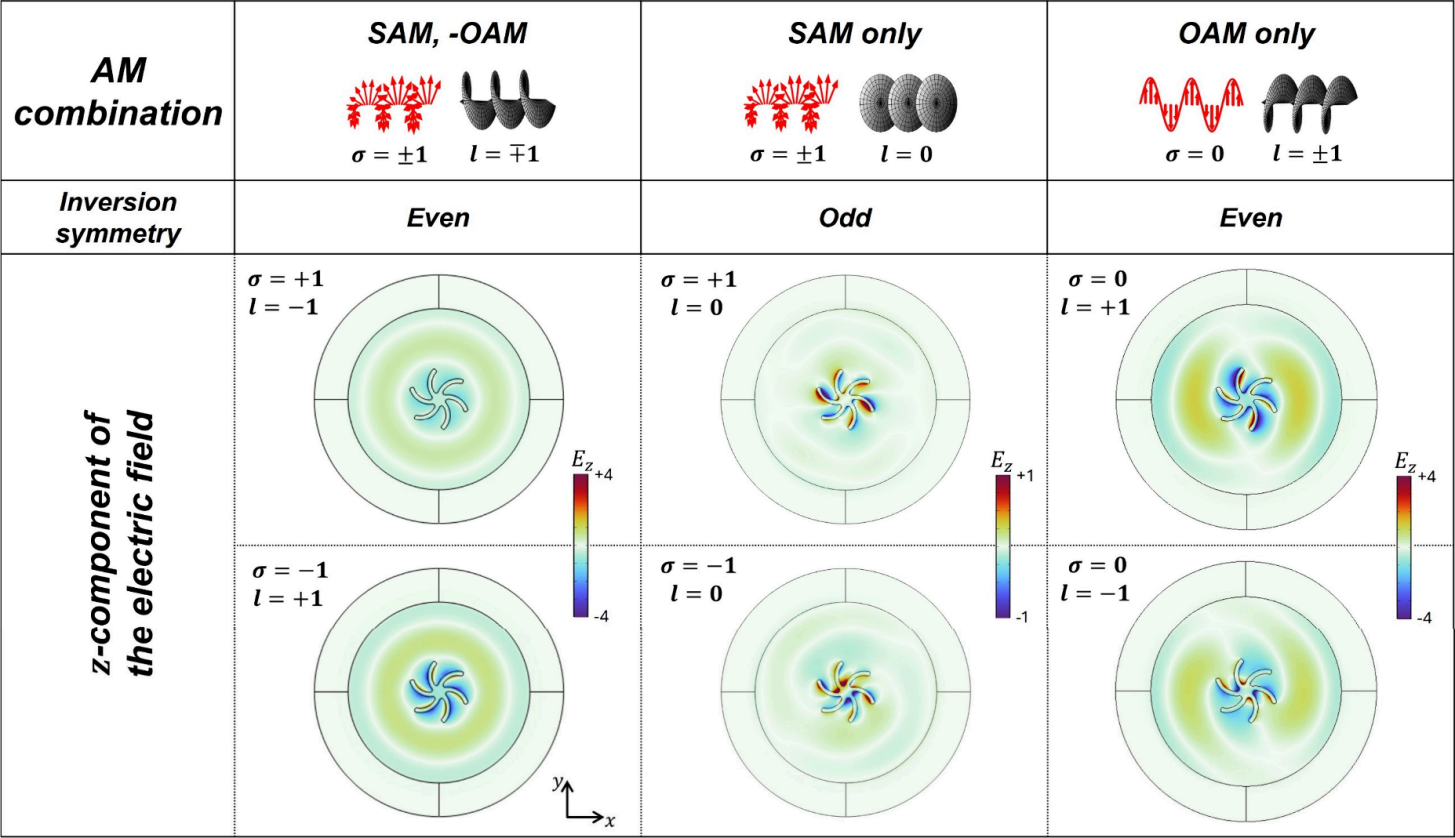
Simulation for the tightly focused beam and shuriken structure. Simulated *z*-component of the electric field plotted on top of a RH shuriken structure for varying combinations of SAM and OAM (λ = 590 nm). The helicity parameter σ and topological charge *l* are indicated in the upper left corner of each panel. When OAM is present, the mode symmetry is even under spatial inversion, corresponding to the excitation of higher-order modes. For SAM only, the mode symmetry is odd under spatial inversion, indicative of the dipole mode excitation.

Finally, the contrast between the weakly and tightly focused regimes underscores the role of collective interactions in shaping the chiroptical response of the metasurface. While Rayleigh anomalies and surface lattice resonances (SLRs) are not expected at 565 nm – given the (1,0) and (1,1) diffraction edges occur above 800 nm for a 720 nm-periodic array on a polycarbonate substrate (*n* ≈ 1.58) – evanescent near-field coupling between neighboring structures can give rise to subdiffraction collective resonances. These interactions are highly sensitive to the coherence area of the illumination and are progressively suppressed with increasing NA. Electromagnetic simulations under paraxial plane-wave excitation show enhanced field intensities in the gaps between adjacent motifs, confirming the presence of interstructure coupling under weak focusing (Figure S9), whereas this coupling disappears for a single structure (Figure S10).

In conclusion, we have demonstrated a real-time, all-optical method for modulating optical activity in chiral metasurfaces using structured light that combines spin and orbital angular momentum. While the observed optical activity originates from the spin (i.e., circular polarization) of the beam, the orbital component does not directly contribute to the chiroptical response. Instead, the presence of orbital angular momentum serves to structure the electromagnetic field, enabling access to dark multipolar modes through enhanced field gradients under tight focusing. This noninvasive mechanism bypasses the limitations of slower, energy-intensive thermal or mechanical modulation strategies^2,21^ and, importantly, it requires no physical reconfiguration of the metasurface. As such, it provides a simple, scalable, and flexible route toward reconfigurable chiral photonic systems.

This technique opens new possibilities for reconfigurable nanophotonic systems, such as those based on optically tunable chiral meta-molecules,^22^ with potential applications in quantum communication, biosensing and optical logic. Future efforts will explore spectral tunability, multiplexed polarization control and integration with active materials for fully programmable metasurfaces. The findings of this work could also be applied to other emerging chiral materials, such as chiral nanocrystals or plasmonic hybrid nanostructures.^23–25^ By establishing structured light as a dynamic optical actuator, this work lays the foundation for high-speed, adaptive chiroptical technologies.

## Supplementary Material



## Notes

Sample fabrication, experimental and computational methods, beam profiles, spatial and structure variation in experimental dichroism, theoretical derivation of focused fields, simulations of weakly and tightly focused beams in free space, simulations of weakly and tightly focused beams shifted with respect to the nanostructure, and simulations of periodic and nonperiodic structures with plane-wave illumination (PDF)
