# Supplementary material for: Optical Activity Modulation in Chiral Metasurfaces via Structured Light
Source: Nano Lett. 2025 Aug 5;25(32):12393–8. doi: 10.1021/acs.nanolett.5c03044 (PMC12356053; doi:10.1021/acs.nanolett.5c03044)
Supplement: Supplementary file 1 [file nl5c03044_si_001.pdf]

Supplementary Information for Optical Activity Modulation in Chiral Metasurfaces via Structured Light

Paula L. Lalaguna[1*], Shun Hashiyada[2*], Nikolaj Gadegaard[3], Jörg B. Götte[4], Stephen M. Barnett[4], Kayn A. Forbes[5], Yoshito Y. Tanaka[2*] & Malcolm Kadodwala[1*]

[1] School of Chemistry, University of Glasgow, Glasgow G12 8QQ, United Kingdom.

[2] Research Institute for Electronic Science, Hokkaido University, Sapporo, Hokkaido 001-0021, Japan.

[3] School of Engineering, Rankine Building, Glasgow G12 8LT, United Kingdom.

[4] SUPA, School of Physics and Astronomy, University of Glasgow, Glasgow G12 8QQ, United Kingdom.

[5] School of Chemistry, University of East Anglia, Norwich Research Park, Norwich NR4 7TJ, United Kingdom.

*Email correspondence:

Paula.LabordaLalaguna@glasgow.ac.uk

shun.hashiyada@es.hokudai.ac.jp

ytanaka@es.hokudai.ac.jp

Malcolm.Kadodwala@glasgow.ac.uk

Table of Contents

Sample fabrication 2

Atomic force microscopy 2

Optical-rotatory dispersion 2

Dichroism measurements under weak and tight focusing 2

Beam spot size calculation 2

Numerical simulations with circularly-polarized plane waves and periodic structure 3

Numerical simulations for weakly- and tightly-focused structured light 3

Supplementary figures 9

References 25

Sample fabrication

The chiral shuriken-shaped nanoindentations were fabricated using an injection moulding technique as described in detail by Gadegaard *et al.*[5] An injection moulding machine (ENGEL) was used to fabricate the nanoindentations first in a polycarbonate substrate.[6] The nanoindentations in the chiral shuriken have a depth of 80nm and a pitch of 720nm.

Prior to gold evaporation, the substrate slides were cleaned with IPA and dried with N_2 gas. 100nm of gold were then evaporated onto the clean slides using an e-beam evaporator (Plassys MEB550S) at a rate of 0.3 nm/s. While the evaporation process takes place, the gold takes the shape of the nanoindentations. More details about the plasmonic behaviour of these chiral shuriken-shaped nanoindentations can be found in Karimullah *et al.*[6]

Atomic force microscopy

Atomic force miscroscopy (AFM) measurements were undertaken to characterize the chiral shuriken-shaped nanoindentations (Figure 1 of the main manuscript). AFM was done using a Dimension Icon Atomic Force Microscopy System with ScanAsyst and a ScanAsyst-Air-HPI tip, made of silicon, and the images were processed using Gwyddion software.

Optical-rotatory dispersion

Optical-rotatory dispersion (ORD) was measured using a home-built polarization microscope system equipped with a 500-9000nm stabilized light source (Thorlabs). In this case, the beam is Gaussian ($l = 0$) and it becomes linearly-polarized ($\sigma = 0$). The beam is focused on the sample using a 10X objective (NA=0.3) and the spectra are measured in a reflection geometry using a spectrometer (Ocean optics USB4000). The intensity of the light is measured using an analyzer at four different angles to obtain an ORD spectra.

Dichroism measurements under weak and tight focusing

To experimentally characterize the dichroism of the samples, we constructed a system consisting mainly of a wavelength-tunable laser (CW, 500 to 750 nm), a photoelastic modulator (PEM), optical SAM–OAM conversion device (q-plate), a polarizer, an objective lens, an xyz piezo stage, and a photodetector. The PEM and q-plate were used to generate a Laguerre-Gaussian beam carrying both OAM and SAM. For comparative studies requiring circularly-polarized light with SAM only, the q-plate was removed from the optical path. The laser beam was focused on the samples placed on the piezo stage using the objective lenses (NA=0.3 or 0.95). Reflected light from the samples was collected by the same objective lens and directed to the photodetector. The extinction spectra of the samples were obtained by calculating $A(r,\lambda) = (R_{ref}(\lambda) - R_{sample}(r,\lambda))/R_{ref}(\lambda)$, in which R_{sample} and R_{ref} represent the intensities of the reflected light at the shuriken structures and plain gold (off the structures), respectively. More details on the experimental set-up can be found in [7].

Beam spot size calculation

The intensity beam profile at an arbitrary wavelength ($\lambda = 600$nm) for the Gaussian ($l = 0$) beam was fitted to the intensity function

$$I(r) = |(E(r)|^2 = Ae^{-\frac{2(r-r_0)^2}{w_0^2}} \tag{1}$$

from which the beam waist w_0 can be obtained.[8] The beam spot size was defined as $2w_0$. The beam profiles for weak and tight focusing can be seen in **Figure S2** and **Figure S3**.

Numerical simulations with circularly-polarized plane waves and periodic structure

Additional simulations were performed using ports and circularly-polarized plane waves to understand the effect of the periodic array in the optical characteristics for SAM only. The model used for these simulations can be found in **Figure S8**. This includes periodic ports, input port 1 placed above the nanostructure and the output port 2 under the substrate. To account for the periodicity of the structure, the width of the gold layer is set to 720 nm and the model includes periodic boundary conditions (Floquet periodicity) in the x and y directions. The size of the mesh elements in the gold layer span the range 0.44-25 nm. The refractive indices of gold and polycarbonate were taken from references [9] and [10], respectively. Perfectly-matched layers (PMLs) are placed above and below the input and output ports, respectively.

Numerical simulations for weakly- and tightly-focused structured light

Numerical simulations were performed using COMSOL Multiphysics (version 6.2), the Wave Optics module and electromagnetic frequency domain. The simulations were performed using a focused beam and a single shuriken structure. The focused fields were derived following the diffraction theory developed by Richards and Wolf.[11,12] As shown in **Figure S11**, the electric field vector rotates by an angle θ when the beam at a distance f after the lens, where f is the focal length of the lens. The maximum angle of rotation is determined by θ_{max}, which is derived from the numerical aperture NA of the objective lens:

$$\text{NA} = n \sin\theta_{max}. \tag{2}$$

The rotation of the incident electric field $\mathbf{E_{inc}}$ by the lens can be written as[3]

$$\begin{aligned}\mathbf{E}_\infty(\theta,\phi) &= \left[\mathbf{E_{inc}}(\theta,\phi)\cdot\begin{pmatrix}-\sin\phi\\ \cos\phi\\ 0\end{pmatrix}\right]\begin{pmatrix}-\sin\phi\\ \cos\phi\\ 0\end{pmatrix}\sqrt{\frac{\mathrm{n_1}}{n_2}}\,(\cos\theta)^{\frac{1}{2}} + \\ &+\left[\mathbf{E_{inc}}(\theta,\phi)\cdot\begin{pmatrix}\cos\phi\\ \sin\phi\\ 0\end{pmatrix}\right]\begin{pmatrix}\cos\theta\cos\phi\\ \cos\theta\sin\phi\\ -\sin\theta\end{pmatrix}\sqrt{\frac{\mathrm{n_1}}{n_2}}\,(\cos\theta)^{\frac{1}{2}}.\end{aligned} \tag{3}$$

In equation (3), n_1 and n_2 are the refractive indices before and after the lens, respectively. In our case, $n_1 = n_2 = 1$ for all the calculations that follow. For an x-polarized beam, equation (3) becomes

$$\begin{aligned}&\mathbf{E}_\infty(\theta,\phi) = \\ &= |\mathbf{E_{inc}}|(\cos\theta)^{1/2}\frac{1}{2}\begin{bmatrix}(1+\cos\theta)-(1-\cos\theta)\cos(2\phi)\\ -(1-\cos\theta)\sin(2\phi)\\ -2\sin\theta\cos\phi\end{bmatrix}.\end{aligned} \tag{4}$$

For a y-polarized beam, equation (3) becomes

$$\mathbf{E}_\infty(\theta,\phi) =$$

$$= |\mathbf{E_{inc}}|(\cos\theta)^{1/2}\frac{1}{2}\begin{bmatrix} -(1-\cos\theta)\sin(2\phi) \\ (1+\cos\theta)+(1-\cos\theta)\cos(2\phi) \\ -2\sin\theta\sin\phi \end{bmatrix}. \tag{5}$$

The electric field near the focus is calculated using the expression[3]

$$\mathbf{E_f}(\rho,\varphi,z) = -\frac{ikfe^{-ikf}}{2\pi}\int_0^{\theta_{max}}\int_0^{2\pi}\mathbf{E}_\infty(\theta,\phi)\, e^{ikz\cos\theta}e^{ik\rho\sin\theta\cos(\phi-\varphi)}\sin\theta\, d\phi d\theta, \tag{6}$$

where $x = \rho\cos\varphi$ and $y = \rho\sin\varphi$ are the transverse components of the electric field near the focus. Equation (6) is used to calculate the electric field near the focus of an arbitrary beam focused with a lens with numerical aperture NA, in equation (2), focal length f and $0 < \theta_{max} < \pi/2$.[3]

For the SAM only case, the beam is Gaussian and circularly polarized. Thus, the incident electric field amplitude in equations (4) and (5) is described with the Gaussian function

$$|\mathbf{E_{inc}}|_{\mathbf{HG00}} = E_0 e^{-(x_\infty+y_\infty)/w_0^2} = E_0 f_w(\theta). \tag{7}$$

$f_w(\theta)$ denotes the apodization function[3]

$$f_w(\theta) = e^{-f^2\sin^2\theta/w_0^2}. \tag{8}$$

Thus, the x- and y-polarized Gaussian beams are given by

$$\mathbf{E}_{\mathbf{HG00}}^{x\mathbf{-polarized}}(\rho,\varphi,z) = -\frac{ikf}{2}E_0 e^{-ikf}\begin{bmatrix} I_{00}+I_{02}\cos(2\varphi) \\ I_{02}\sin(2\varphi) \\ -2iI_{01}\cos(\varphi) \end{bmatrix} \tag{9}$$

$$\mathbf{E}_{\mathbf{HG00}}^{y\mathbf{-polarized}}(\rho,\varphi,z) = -\frac{ikf}{2}E_0 e^{-ikf}\begin{bmatrix} I_{02}\sin(2\varphi) \\ I_{00}-I_{02}\cos(2\varphi) \\ -2iI_{01}\sin(\varphi) \end{bmatrix} \tag{10}$$

where the following abbreviation integrals over θ have been used:[3,11]

$$I_{00}(\rho,z) = \int_0^{\theta_{max}} f_w(\theta)(\cos\theta)^{\frac{1}{2}}\sin\theta\,(1+\cos\theta)J_0(k\rho\sin\theta)e^{ikz\cos\theta}d\theta, \tag{11}$$

$$I_{01}(\rho,z) = \int_0^{\theta_{max}} f_w(\theta)(\cos\theta)^{\frac{1}{2}}\sin^2\theta\, J_1(k\rho\sin\theta)e^{ikz\cos\theta}d\theta, \tag{12}$$

$$I_{02}(\rho,z) = \int_0^{\theta_{max}} f_w(\theta)(\cos\theta)^{\frac{1}{2}}\sin\theta\,(1-\cos\theta)J_2(k\rho\sin\theta)e^{ikz\cos\theta}d\theta, \tag{13}$$

$$I_{10}(\rho,z) = \int_0^{\theta_{max}} f_w(\theta)(\cos\theta)^{\frac{1}{2}}\sin^3\theta\,J_0(k\rho\sin\theta)e^{ikz\cos\theta}d\theta, \tag{14}$$

$$I_{11}(\rho,z) = \int_0^{\theta_{max}} f_w(\theta)(\cos\theta)^{\frac{1}{2}}\sin^2\theta\,(1+3\cos\theta)J_1(k\rho\sin\theta)e^{ikz\cos\theta}d\theta, \tag{15}$$

$$I_{12}(\rho,z) = \int_0^{\theta_{max}} f_w(\theta)(\cos\theta)^{\frac{1}{2}}\sin^2\theta\,(1-\cos\theta)J_1(k\rho\sin\theta)e^{ikz\cos\theta}d\theta, \tag{16}$$

$$I_{13}(\rho,z) = \int_0^{\theta_{max}} f_w(\theta)(\cos\theta)^{\frac{1}{2}}\sin^3\theta\,J_2(k\rho\sin\theta)e^{ikz\cos\theta}d\theta, \tag{17}$$

$$I_{14}(\rho,z) = \int_0^{\theta_{max}} f_w(\theta)(\cos\theta)^{\frac{1}{2}}\sin^2\theta\,(1-\cos\theta)J_3(k\rho\sin\theta)e^{ikz\cos\theta}d\theta. \tag{18}$$

The circularly-polarized Gaussian beam is given by

$$\mathbf{E}_{\mathbf{HG00}}^{\sigma=\pm1} = \frac{1}{\sqrt{2}}\left(\mathbf{E}_{\mathbf{HG00}}^{x-\mathbf{polarized}} \pm i\mathbf{E}_{\mathbf{HG00}}^{y-\mathbf{polarized}}\right), \tag{19}$$

thus, equation (19) becomes

$$\mathbf{E}_{\mathbf{HG00}}^{\sigma=\pm1}(\rho,\varphi,z) = -\frac{1}{\sqrt{2}}\frac{ikf}{2}E_0e^{-ikf}\begin{bmatrix} I_{00}+I_{02}e^{\pm i2\varphi} \\ \pm iI_{00} \mp iI_{02}e^{\pm i2\varphi} \\ -2iI_{01}e^{\pm i\varphi}\end{bmatrix} =$$

$$= -\frac{1}{\sqrt{2}}\frac{ikf}{2}E_0e^{-ikf}\left(I_{00}\begin{bmatrix}1\\ \pm i\\ 0\end{bmatrix} + I_{01}e^{\pm i\varphi}\begin{bmatrix}0\\ 0\\ -2i\end{bmatrix} + I_{02}e^{\pm i2\varphi}\begin{bmatrix}1\\ \mp i\\ 0\end{bmatrix}\right). \tag{20}$$

As seen in equation (20), the focused circularly-polarized Gaussian beam contains contributions for SAM only ($\sigma = \pm1, l = 0$) which is the dominant term under weak focusing. For tighter focusing, the second and third terms, corresponding to OAM only ($\sigma = 0, l = \pm1$) and SAM-OAM ($\sigma = \mp1, l = \pm2$) terms respectively.

The circularly-polarized Laguerre-Gaussian beam with antiparallel SAM-OAM ($\sigma = \mp1$, $l = \pm1$) can be constructed from a combination of Hermite-Gaussian (HG) modes. The amplitude of HG10 and HG01 modes is given by

$$|\mathbf{E_{inc}}|_{\mathbf{HG10}} = E_0(2x_\infty/w_0)e^{-\frac{x_\infty+y_\infty}{w_0^2}} = (2E_0f/w_0)\sin\theta\cos\phi\, f_w(\theta), \tag{21}$$

$$|\mathbf{E}_{\mathbf{inc}}|_{\mathbf{HG01}} = E_0(2y_\infty/w_0)e^{-(x_\infty+y_\infty)/w_0^2} = (2E_0 f/w_0)\sin\theta\sin\phi\, f_w(\theta). \tag{22}$$

Therefore, the focused fields for x- and y-polarized HG modes are given by

$$\mathbf{E}_{\mathbf{HG10}}^{x\mathbf{-polarized}}(\rho,\varphi,z) = -\frac{ikf^2}{2w_0}E_0 e^{-ikf}\begin{bmatrix} iI_{11}\cos\varphi + iI_{14}\cos(3\varphi) \\ iI_{14}\sin(3\varphi) - iI_{12}\sin\varphi \\ -2I_{10} + 2I_{13}\cos(2\varphi) \end{bmatrix}, \tag{23}$$

$$\mathbf{E}_{\mathbf{HG01}}^{x\mathbf{-polarized}}(\rho,\varphi,z) = -\frac{ikf^2}{2w_0}E_0 e^{-ikf}\begin{bmatrix} i(I_{11} + 2I_{12})\sin\varphi + iI_{14}\sin(3\varphi) \\ -iI_{12}\cos\varphi - iI_{14}\cos(3\varphi) \\ 2I_{13}\sin(2\varphi) \end{bmatrix}, \tag{24}$$

$$\mathbf{E}_{\mathbf{HG10}}^{y\mathbf{-polarized}}(\rho,\varphi,z) = -\frac{ikf^2}{2w_0}E_0 e^{-ikf}\begin{bmatrix} iI_{14}\sin(3\varphi) - iI_{12}\sin\varphi \\ i(I_{11} + 2I_{12})\cos\varphi - iI_{14}\cos(3\varphi) \\ 2I_{13}\sin(2\varphi) \end{bmatrix}, \tag{25}$$

$$\mathbf{E}_{\mathbf{HG01}}^{y\mathbf{-polarized}}(\rho,\varphi,z) = -\frac{ikf^2}{2w_0}E_0 e^{-ikf}\begin{bmatrix} -iI_{14}\cos(3\varphi) - iI_{12}\cos\varphi \\ iI_{11}\sin\varphi - iI_{14}\sin(3\varphi) \\ -2I_{10} - 2I_{13}\cos(2\varphi) \end{bmatrix}. \tag{26}$$

The x-polarized LG beam ($\sigma = 0, l = \pm 1$) is obtained from a combination of HG modes *via*

$$\mathbf{E}_{l=\pm 1}^{x\mathbf{-polarized}}(\rho,\varphi,z) = \frac{1}{\sqrt{2}}\left(\mathbf{E}_{\mathbf{HG10}}^{x\mathbf{-polarized}} \pm i\mathbf{E}_{\mathbf{HG01}}^{y\mathbf{-polarized}}\right). \tag{27}$$

Therefore, equation (27) becomes

$$\begin{aligned}
&\mathbf{E}_{l=\pm 1}^{x\mathbf{-polarized}}(\rho,\varphi,z) \\
&= -\frac{1}{\sqrt{2}}\frac{ikf^2}{2w_0}E_0 e^{-ikf}\left(\begin{bmatrix} iI_{11}\cos(\varphi) + iI_{14}\cos(3\varphi) \\ iI_{14}\sin(3\varphi) - iI_{12}\sin(\varphi) \\ -2I_{10} + 2I_{13}\cos(2\varphi) \end{bmatrix}\right. \\
&\left.\pm i\begin{bmatrix} i(I_{11} + 2I_{12})\sin\varphi + iI_{14}\sin(3\varphi) \\ -iI_{12}\cos\varphi - iI_{14}\cos(3\varphi) \\ 2I_{13}\sin(2\varphi) \end{bmatrix}\right) = \\
&\propto iI_{11}e^{\pm i\varphi}\begin{bmatrix}1\\0\\0\end{bmatrix} + iI_{14}e^{\pm i3\varphi}\begin{bmatrix}1\\\mp i\\0\end{bmatrix} + iI_{12}\sin\varphi\begin{bmatrix}\pm i\\0\\0\end{bmatrix} + iI_{12}e^{\mp i\varphi}\begin{bmatrix}0\\\mp i\\0\end{bmatrix} \\
&+ 2I_{13}e^{\pm i2\varphi}\begin{bmatrix}0\\0\\1\end{bmatrix} - 2I_{10}\begin{bmatrix}0\\0\\1\end{bmatrix}.
\end{aligned} \tag{28}$$

The circularly-polarized LG beam with antiparallel SAM-OAM beam ($\sigma = \mp 1, l = \pm 1$) is obtained *via*

$$\mathbf{E}_{l=\pm 1}^{\sigma=\mp 1}(\rho,\varphi,z) = \frac{1}{\sqrt{2}}\left(\mathbf{E}_{\mathbf{HG10}}^{x\text{-polarized}} + \mathbf{E}_{\mathbf{HG01}}^{y\text{-polarized}}\right) \mp i\left(-\mathbf{E}_{\mathbf{HG01}}^{x\text{-polarized}} + \mathbf{E}_{\mathbf{HG10}}^{y\text{-polarized}}\right). \tag{29}$$

Therefore, equation (29) becomes

$$\begin{aligned}\mathbf{E}_{l=\pm 1}^{\sigma=\mp 1}(\rho,\varphi,z) &= -\frac{1}{\sqrt{2}}\frac{ikf^2}{2w_0}E_0 e^{-ikf}\left(\begin{bmatrix} i(I_{11}-I_{12})\cos(\varphi) \\ i(I_{11}-I_{12})\sin(\varphi) \\ -4I_{10}\end{bmatrix}\right.\\ &\left.\pm i\begin{bmatrix} i(I_{11}+3I_{12})\sin(\varphi) \\ -i(I_{11}+3I_{12})\cos\varphi \\ 0\end{bmatrix}\right) = \\ &\propto \frac{i\{(1\pm 1)I_{11}-(1\mp 3)I_{12}\}}{2}e^{+i\varphi}\begin{bmatrix}1\\-i\\0\end{bmatrix} \\ &+\frac{i\{(1\mp 1)I_{11}-(1\pm 3)I_{12}\}}{2}e^{-i\varphi}\begin{bmatrix}1\\+i\\0\end{bmatrix} - 4I_{10}\begin{bmatrix}0\\0\\1\end{bmatrix}.\end{aligned} \tag{30}$$

For completeness, the simulations in air were also tested for circularly-polarized LG beam with parallel SAM-OAM beam ($\sigma = \pm 1, l = \pm 1$), which is obtained *via*

$$\mathbf{E}_{l=\pm 1}^{\sigma=\pm 1}(\rho,\varphi,z) = \frac{1}{\sqrt{2}}\left(\mathbf{E}_{\mathbf{HG10}}^{x\text{-polarized}} - \mathbf{E}_{\mathbf{HG01}}^{y\text{-polarized}}\right) \pm i\left(\mathbf{E}_{\mathbf{HG01}}^{x\text{-polarized}} + \mathbf{E}_{\mathbf{HG10}}^{y\text{-polarized}}\right). \tag{31}$$

Therefore, equation (31) becomes

$$\begin{aligned}\mathbf{E}_{l=\pm 1}^{\sigma=\pm 1}(\rho,\varphi,z) &= -\frac{1}{\sqrt{2}}\frac{ikf^2}{2w_0}E_0 e^{-ikf}\left(\begin{bmatrix} i(I_{11}+I_{12})\cos\varphi + 2iI_{14}\cos(3\varphi) \\ -i(I_{11}+I_{12})\cos\varphi + 2iI_{14}\sin(3\varphi) \\ 4I_{13}\cos(2\varphi)\end{bmatrix}\right.\\ &\left.\pm i\begin{bmatrix} i(I_{11}+I_{12})\sin\varphi + 2iI_{14}\sin(3\varphi) \\ i(I_{11}+I_{12})\cos\varphi - 2iI_{14}\cos(3\varphi) \\ 4I_{13}\sin(2\varphi)\end{bmatrix}\right) = \\ &\propto i(I_{11}+I_{12})e^{\pm i\varphi}\begin{bmatrix}1\\ \pm i\\0\end{bmatrix} + 2iI_{14}e^{\pm i3\varphi}\begin{bmatrix}1\\ \mp i\\0\end{bmatrix} + 4I_{13}e^{\pm i2\varphi}\begin{bmatrix}0\\0\\1\end{bmatrix}.\end{aligned} \tag{32}$$

Equation (32) contains the parallel combination of SAM and OAM ($\sigma = \pm 1, l = \pm 1$) and the terms originating from spin-orbit conversion due to antiparallel SAM and OAM ($\sigma = \mp 1, l = \pm 3$) and OAM only ($l = \pm 2$) contributions.

The simulations in air (**Figure S12**) showed the expected behaviour that focusing has on the beam intensity. In particular, for the tightly-focused beam, where longitudinal fields are considerable, the intensity is elongated for the linearly-polarized LG beam in the polarization direction and the antiparallel SAM-OAM combination shows come on-axis intensity as it has been observed in previous studies.[1,2,4]

The interaction of the focused fields with shuriken structures was done using a spherical model (**Figure S13**) with a single shuriken structure, placed at $z = 0$, on top of a polycarbonate substrate. The medium above the structure is air. The simulated model has a radius of 500 nm $+ \lambda/2$ and it is embedded in a perfectly-matched layer (PML) of thickness $\lambda/2$. The size of the mesh elements in the gold layer span the range 0.44-25 nm. The refractive indices of gold and polycarbonate were taken from references [9] and [10], respectively.

The absorption (A_{abs}) and scattering (A_{sc}) cross-sections were extracted from the simulations using the formulas

$$A_{abs} = \frac{1}{I_0} \iiint_V Q \, dV, \tag{33}$$

$$A_{sc} = \frac{1}{I_0} \iint_S \mathbf{S_{sc}} \cdot \mathbf{n} \, dS. \tag{34}$$

I_0 is the intensity of the incident beam, Q the power loss density in the gold layer with volume V, $\mathbf{S_{sc}}$ the Poynting vector of the scattered light and $\mathbf{n}$ an outward unit vector normal to the surface around the gold layer S. The extinction (A_{ext}) is then given by the sum of the absorption and scattering cross-sections:

$$A_{ext} = A_{sc} + A_{abs}. \tag{35}$$

The g-factor in the extinction was then calculated using the formula

$$g_{\text{ext}} = \frac{2(A_{ext}^{\sigma=+1} - A_{ext}^{\sigma=-1})}{(A_{ext}^{\sigma=+1} + A_{ext}^{\sigma=-1})}, \tag{36}$$

Supplementary figures

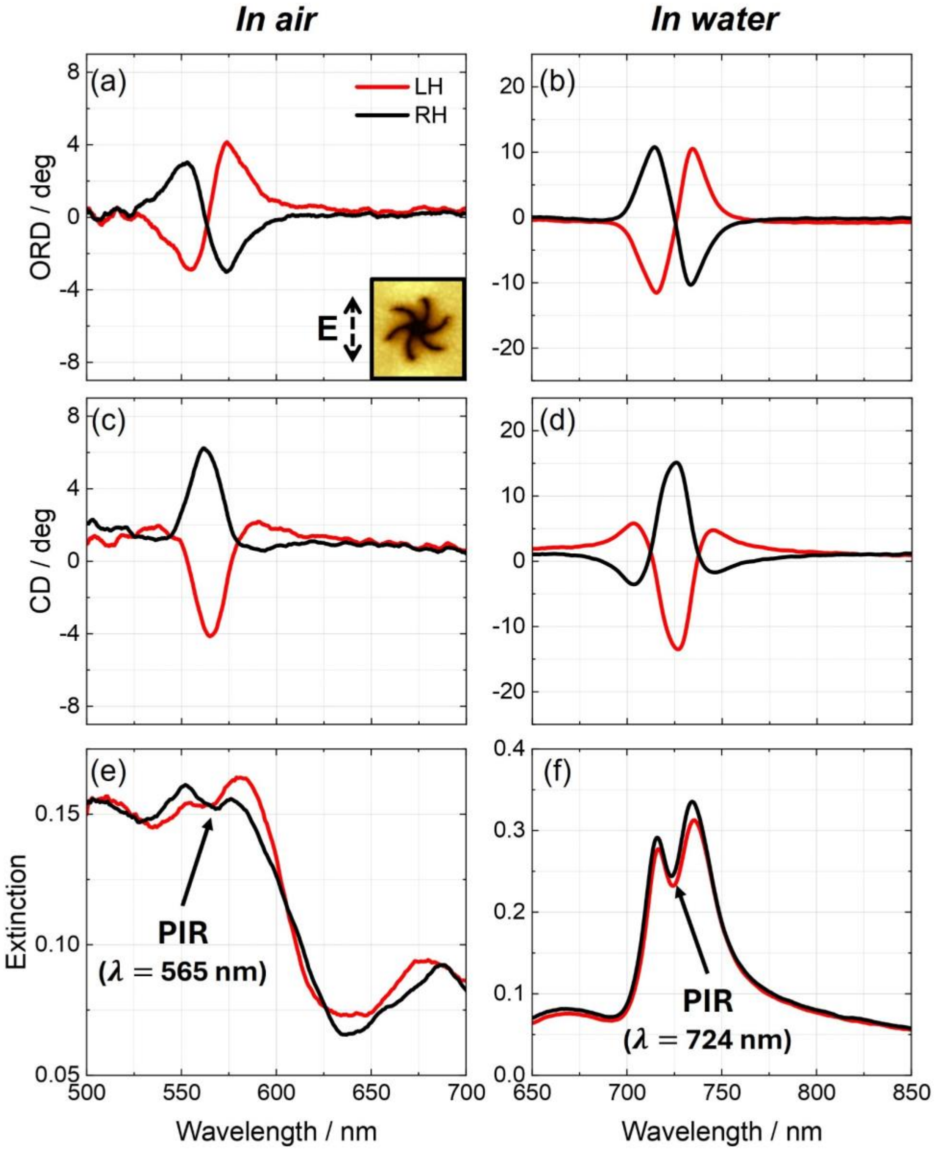

Figure S1: (a,b) ORD spectra of LH (red) and RH (black) shuriken structures for measurements in (a) air and (b) water. (c,d) Calculated CD from ORD using Kramers-Kronig relations from (a,b), respectively. (e,f) Extinction spectra of LH (red) and RH (black) shuriken structures for measurements in (c) air and (d) water. This data was collected using a linearly-polarized Gaussian beam with the incident polarization shown in (a).

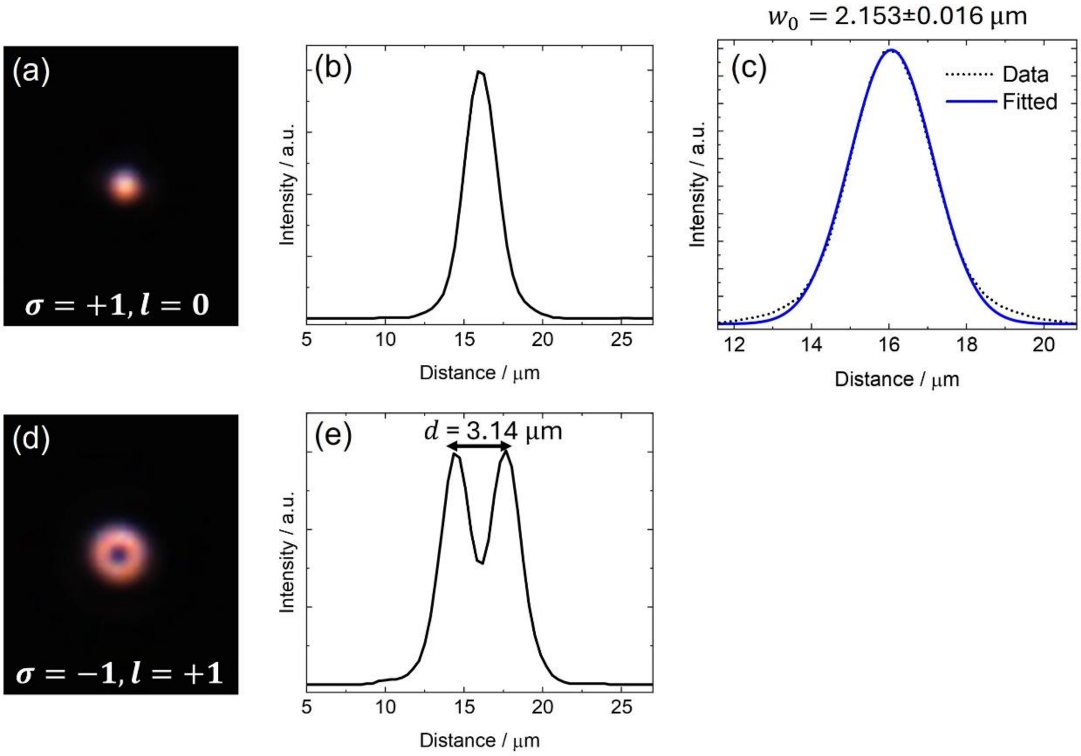

Figure S2: Beam profiles for the weakly-focused beams. (a) Intensity image of the $l = 0$ beam (λ=600nm). (b) Beam profile of the $l = 0$ beam extracted from (a). (c) Beam profile of the $l = 0$ beam (black dotted line) and fitted (blue) to a Gaussian function, from which a beam waist of 2.153±0.016μm is obtained. (d) Intensity image of the $l = 1$ beam (λ=600nm). (e) Beam profile of the $l = 1$ beam extracted from (d), with the peak-to-peak separation indicated as d=3.14μm.

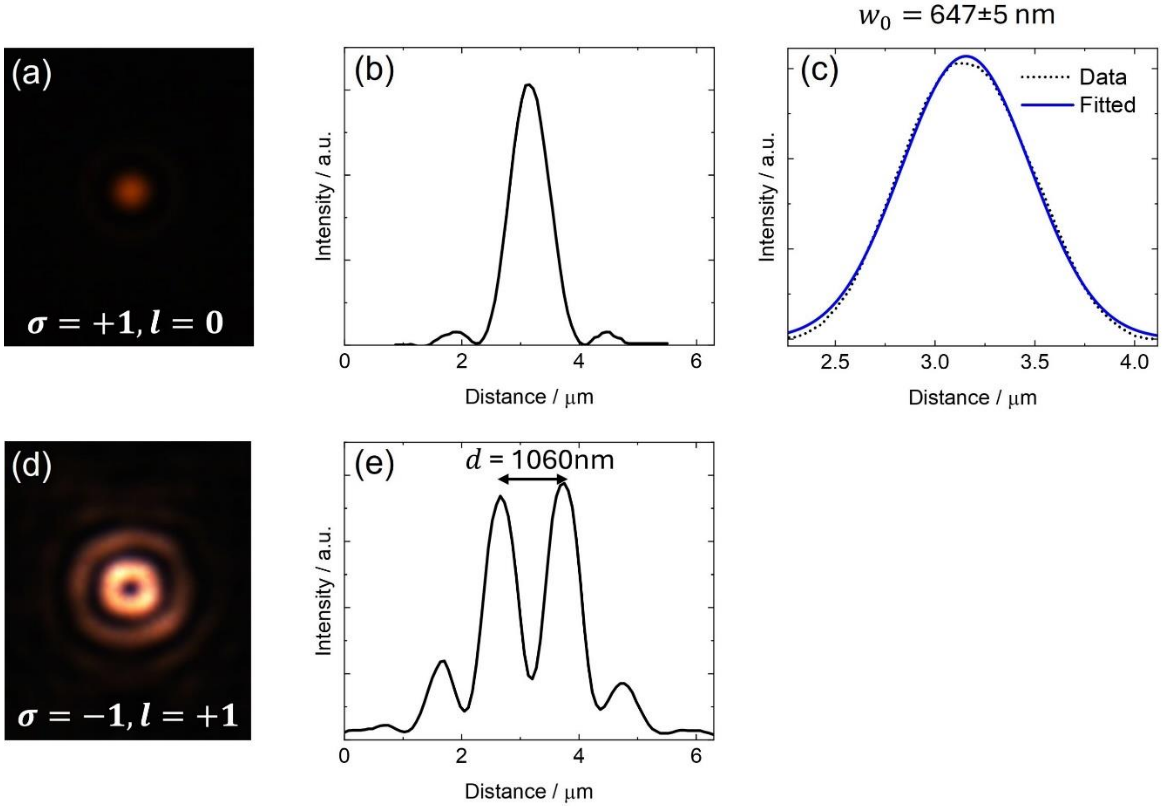

Figure S3: Beam profiles for the tightly-focused beams. (a) Intensity image of the $l = 0$ beam (λ=600nm). (b) Beam profile of the $l = 0$ beam extracted from (a). (c) Beam profile of the $l = 0$ beam (black dotted line) and fitted (blue) to a Gaussian function, from which a beam waist of 647$\pm$5 nm is obtained. (d) Intensity image of the $l = 1$ beam (λ=600nm). (e) Beam profile of the $l = 1$ beam extracted from (d), with the peak-to-peak separation indicated as d=1060 nm.

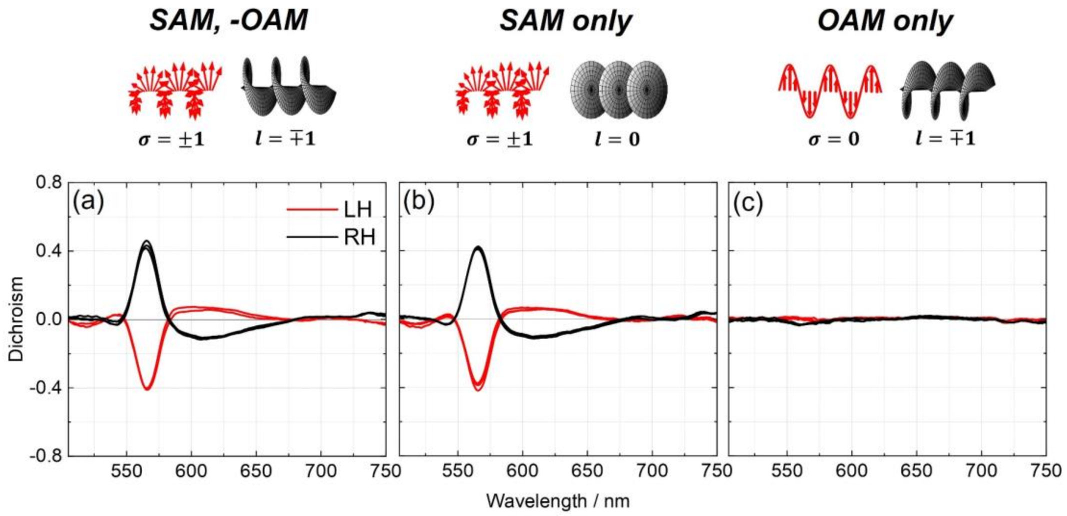

Figure S4: Spatial variation in the dichroism across the 3 random sample positions for the weakly-focused beams in LH (red) and RH (black) shuriken-shaped nanoindentations. (a) antiparallel SAM-OAM combination. (b) SAM only. (c) OAM only.

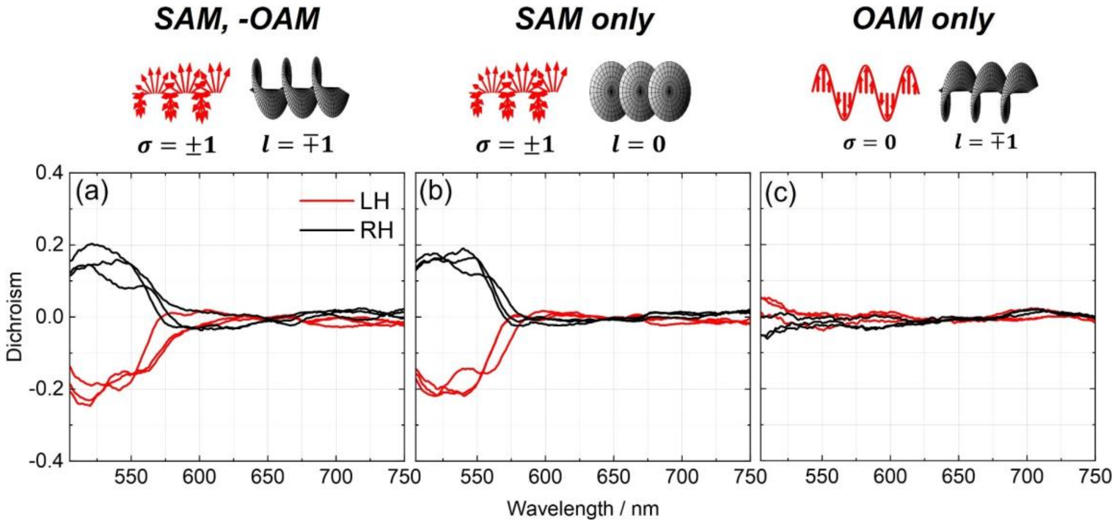

Figure S5: Spatial variation in the dichroism across the 3 random sample positions for the tightly-focused beams in LH (red) and RH (black) shuriken-shaped nanoindentations. (a) antiparallel SAM-OAM combination. (b) SAM only. (c) OAM only.

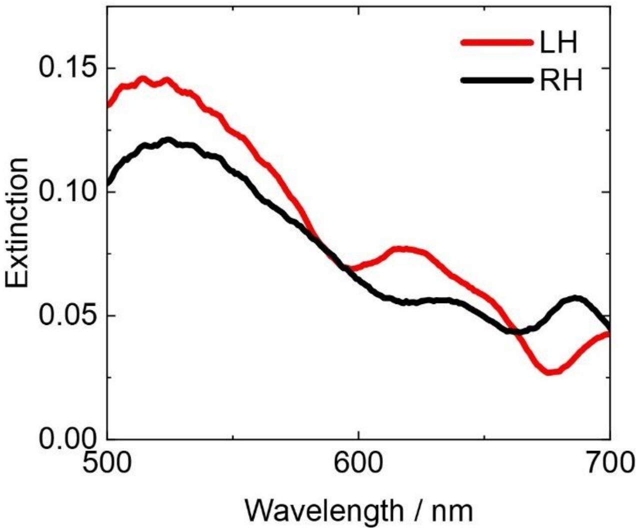

Figure S6: Extinction spectra of LH (red) and RH (black) shuriken structures under tight focusing. The data was collected using a linearly-polarized Gaussian beam. The data shows no PIR under the tightly-focused regime.

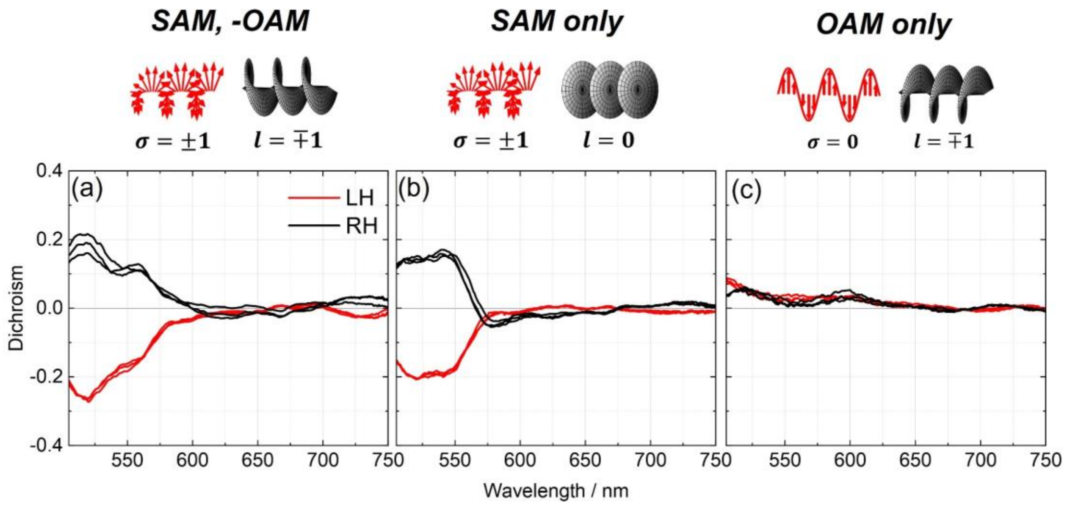

Figure S7: Structure variation in the dichroism collected at 3 different nanostructures for the tightly-focused beams in LH (red) and RH (black) shuriken-shaped nanoindentations. (a) antiparallel SAM-OAM combination. (b) SAM only. (c) OAM only.

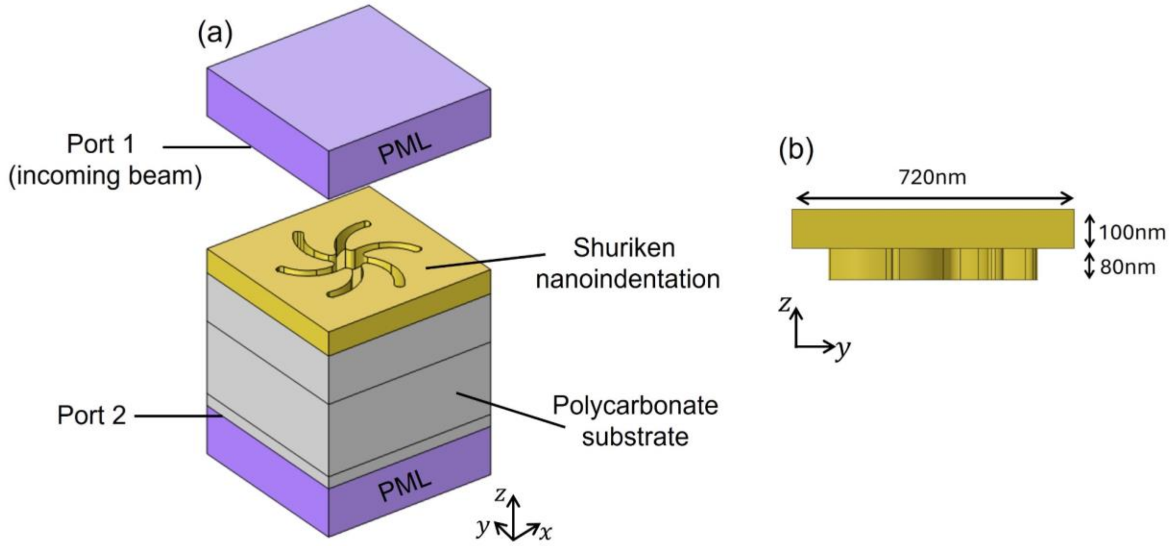

Figure S8: (a) Simulation model for periodic structures. The model includes periodic boundary conditions in the x and y directions (Floquet periodicity) and PMLs (perfectly-matched layers) above and below the input and output ports, respectively. (b) Side view of the gold layer.

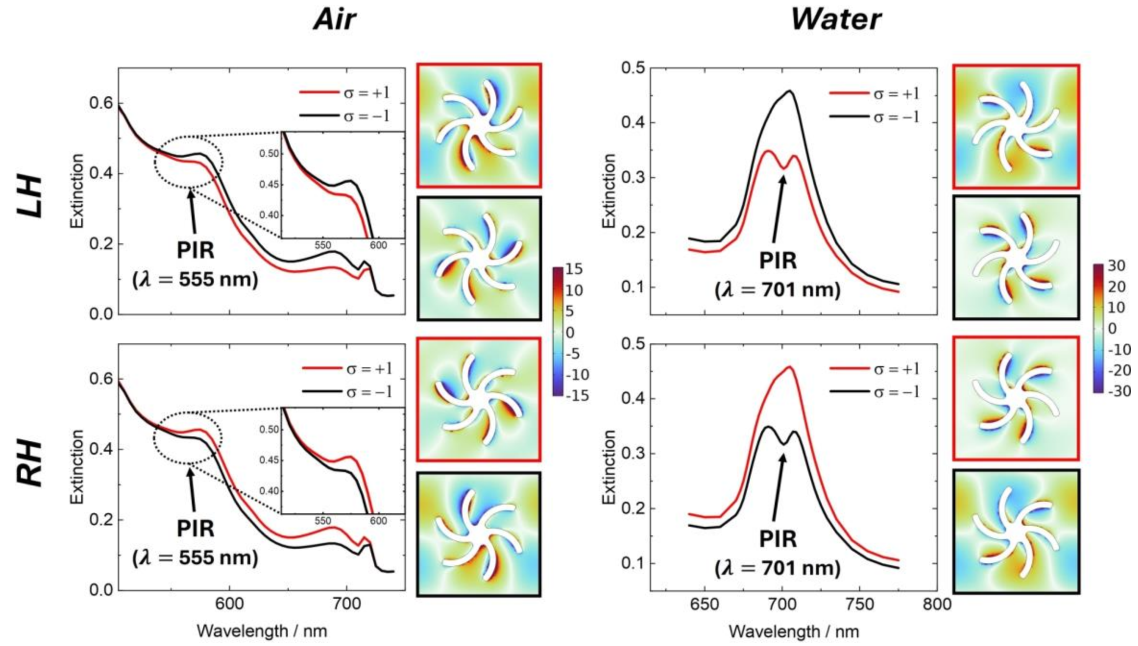

Figure S9: Simulated SAM-dependent extinction for $\sigma = +1$ (red) and $\sigma = -1$ (black) for periodic structures in air and water media. The z-component of the electric field at the PIR wavelength shown in red and black squares for $\sigma = +1$ and $\sigma = -1$ polarizations, respectively.

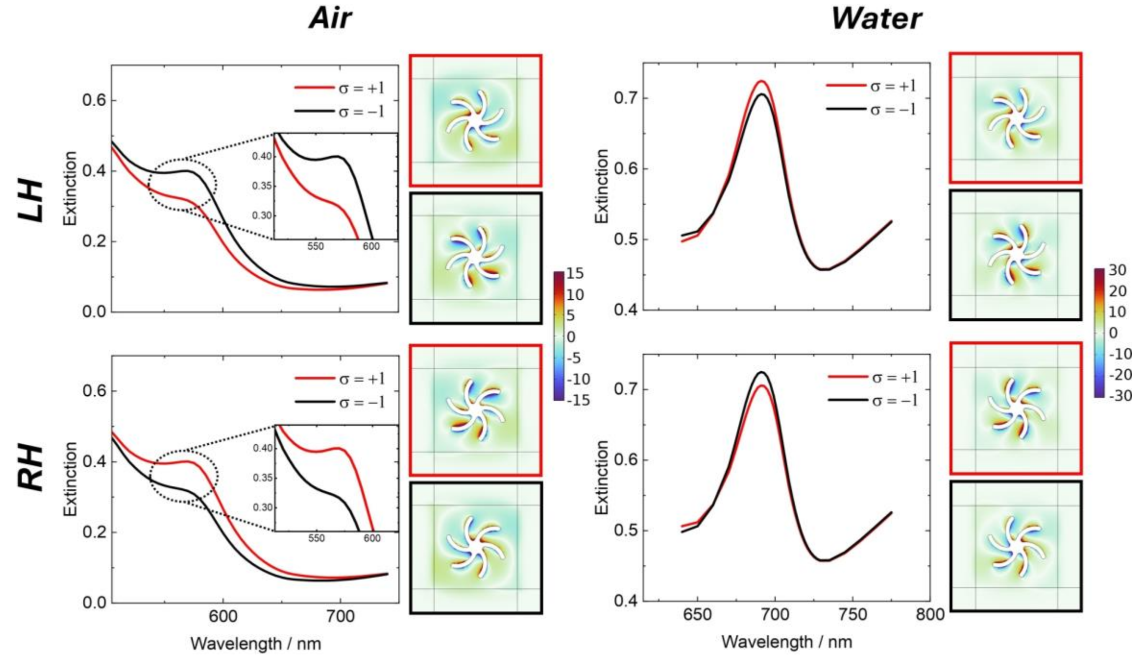

Figure S10: Simulated SAM-dependent extinction for $\sigma = +1$ (red) and $\sigma = -1$ (black) for a single structure in air and water media. The z-component of the electric field at the same wavelengths of Figure S9 is shown in red and black squares for $\sigma = +1$ and $\sigma = -1$ polarizations, respectively.

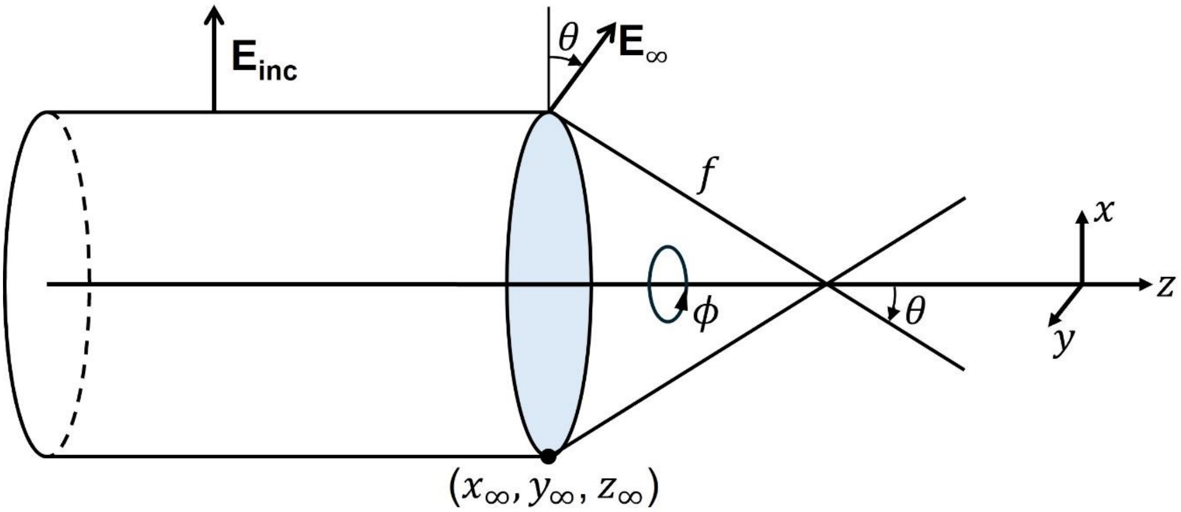

Figure S11: Electric field rotation by lens focusing (the lens is depicted as a blue surface). In this example, the incoming electric field $\mathbf{E_{inc}}$ oscillates along the x direction, and it is rotated by an angle θ by the lens to give the rotated electric field at the lens surface, $\mathbf{E}_\infty$. The focal length of the lens is f, which defines the radius of the cone for the focused fields. $(x_\infty, y_\infty, z_\infty)$ denotes an arbitrary point at the lens surface.[3]

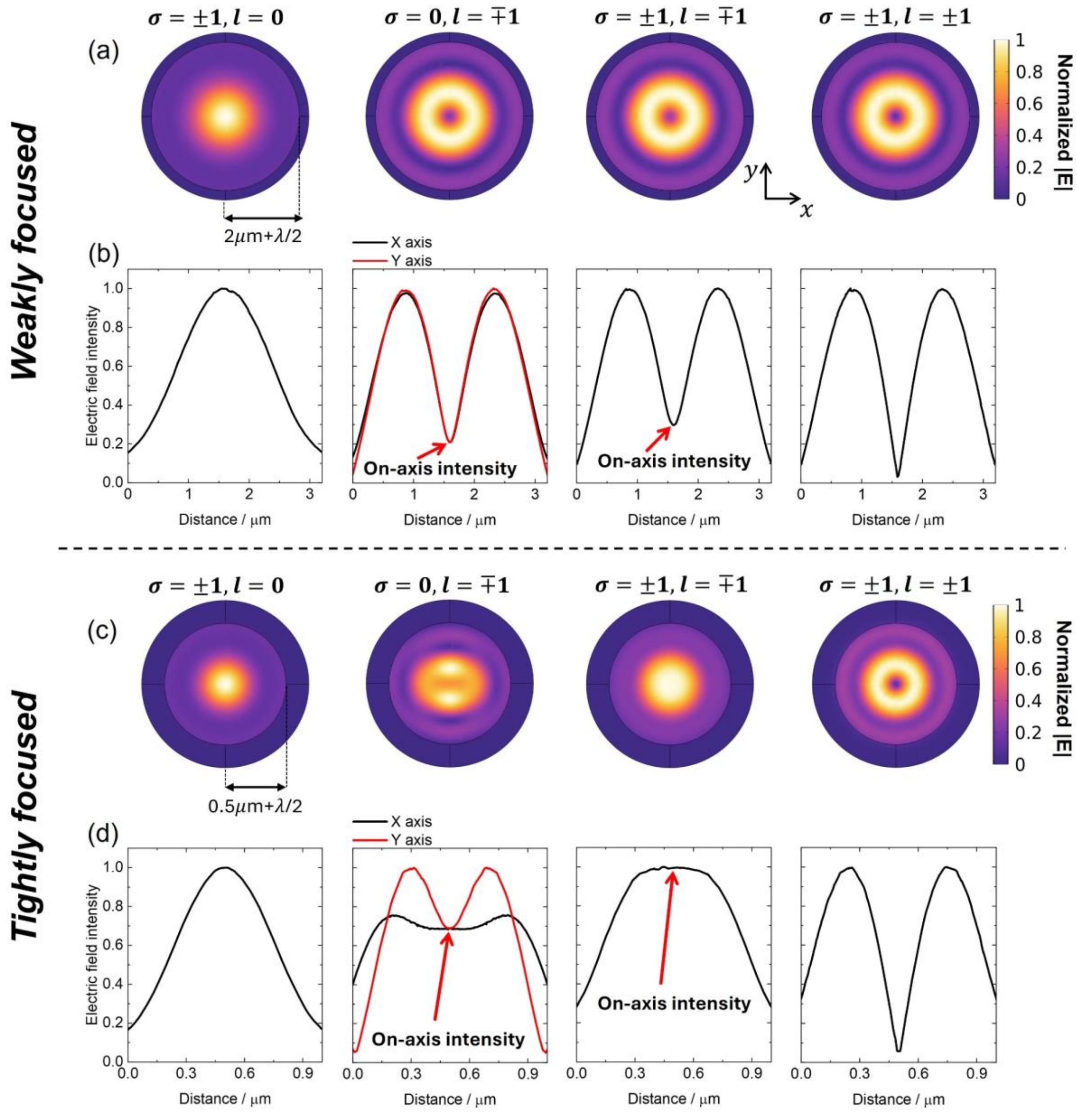

Figure S12: Simulations in air for (a,b) weakly-focused (NA=0.3) and (c,d) tightly-focused (NA=0.95) beams. The simulated beams shown here are circularly-polarized Gaussian ($\sigma = \pm 1$, $l = 0$), linearly-polarized (in the x direction) LG beams ($\sigma = 0$, $l = \mp 1$) and circularly-polarized LG beams with antiparallel ($\sigma = \pm 1$, $l = \mp 1$) and parallel ($\sigma = \pm 1$, $l = \pm 1$) SAM and OAM. (a,c) 2D plot of the electric field intensity. (b,d) 1D plot of the 2D plot shown above. $\lambda = 600$ nm. The simulations show the expected behaviour that focusing and increase in longitudinal fields have on the electric field intensity of structured beams. These are, specifically, on-axis intensity for the antiparallel SAM-OAM case but none in the parallel case.[1,2] Additionally, it can be seen that the intensity of the linearly-polarized LG beam is not evenly distributed and the intensity is elongated in the polarization direction (the x axis), in agreement with previous studies.[4]

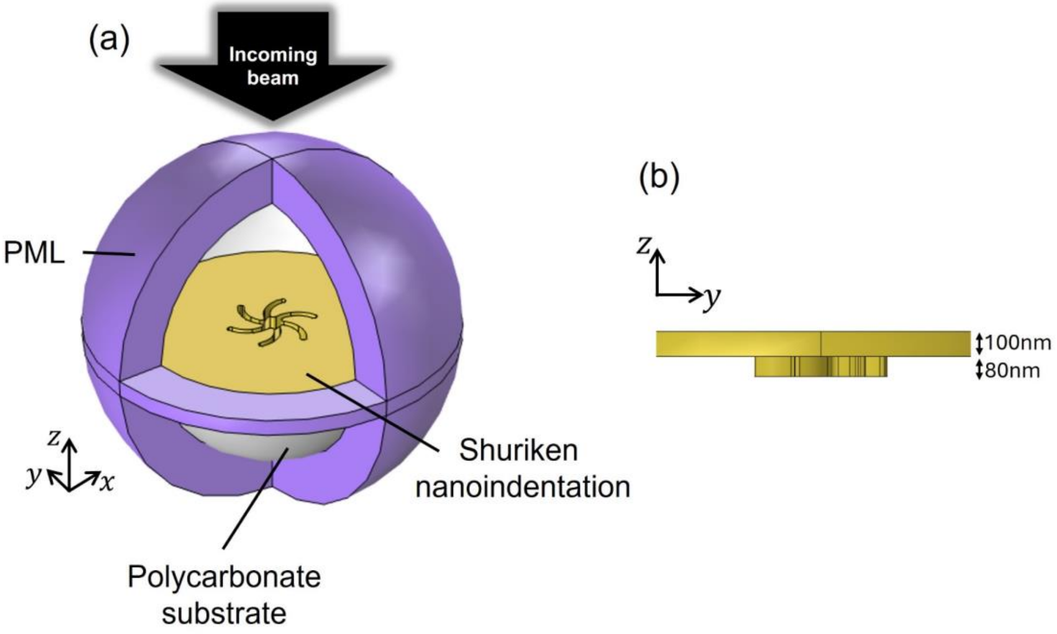

Figure S13: (a) Simulation model containing a single gold shuriken-shaped nanoindentation. The nanoindentation can be left- or right-handed (only the right-handed is shown). Above the shuriken structure there is an air domain and below a polycarbonate substrate. (b) Side view of the gold layer. The beam propagates in the negative z direction.

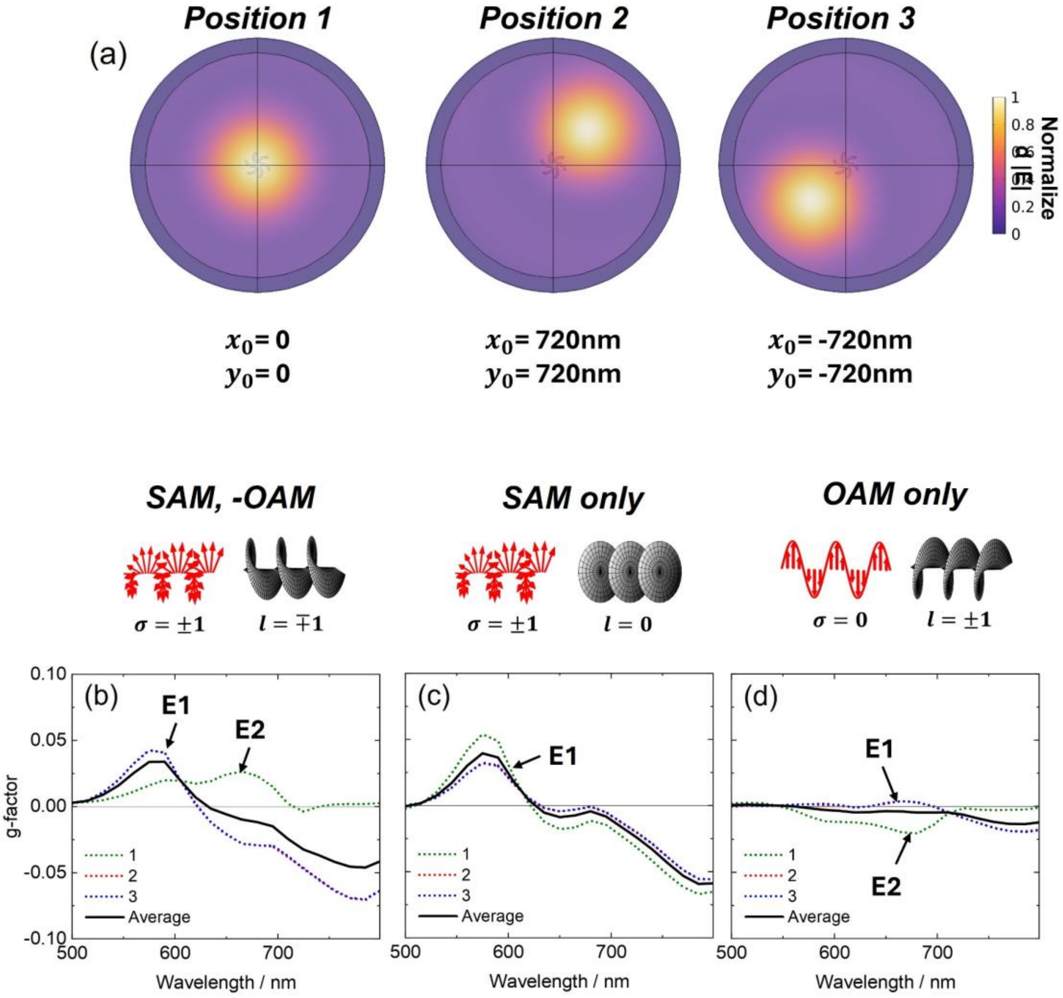

Figure S14: (a) Simulated beam positions with respect to the structure. Position 1: beam centred on the structure. Position 2 (Position 3): the beam has been shifted by $x_0 = 720$ nm ($x_0 = -720$ nm) in the x direction and $y_0 = 720$ nm ($y_0 = -720$ nm) in the y direction. (b-d) Simulated g-factor in the extinction of the RH structure for positions 1 (dotted green), 2 (dotted red) and 3 (dotted blue) with the averaged g-factor shown in black for (b) antiparallel SAM-OAM, (c) SAM only and (d) OAM only. E1: electric dipole excitation. E2: electric quadrupole excitation. For (b) and (c), the averaged g-factor is similar due to the dipole-driven dichroism for the structures off the beam centres in positions 2 and 3 (Figure S16).

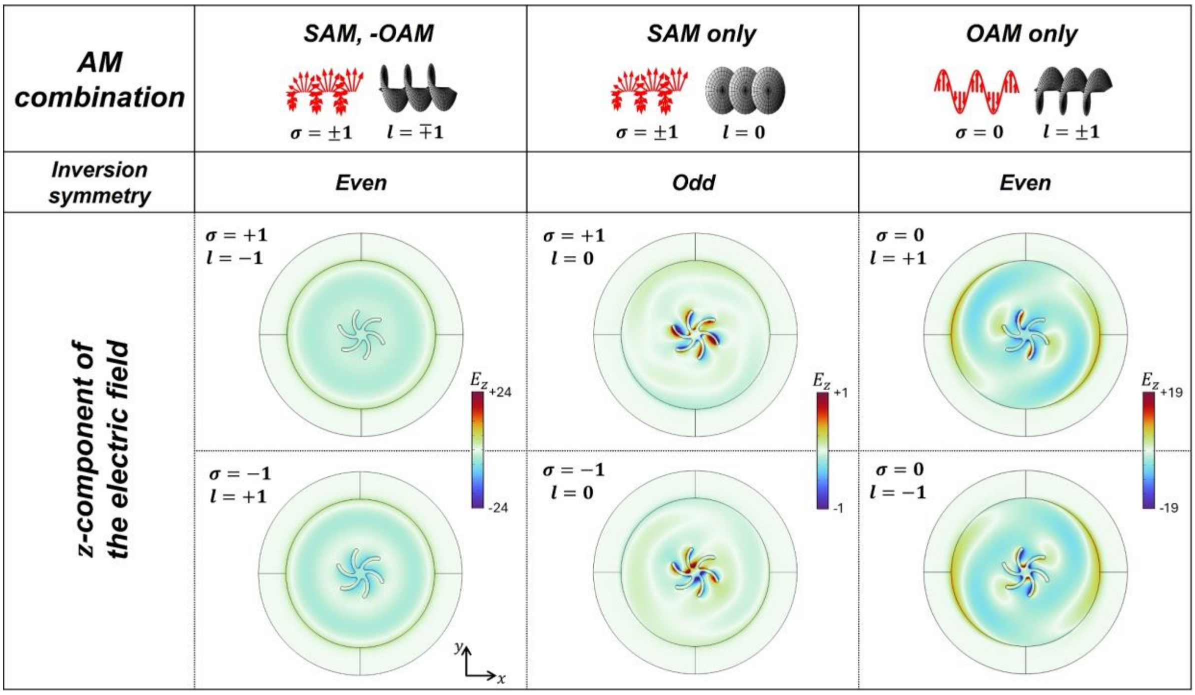

Figure S15: Simulated z-component of the electric field plotted when the weakly-focused beam centred on the structure (position 1 of Figure S14). The field is plotted on top of a RH shuriken structure for varying combinations of SAM and OAM ($\lambda = 590$ nm). The helicity parameter σ and topological charge l are indicated in the upper left corner of each panel.

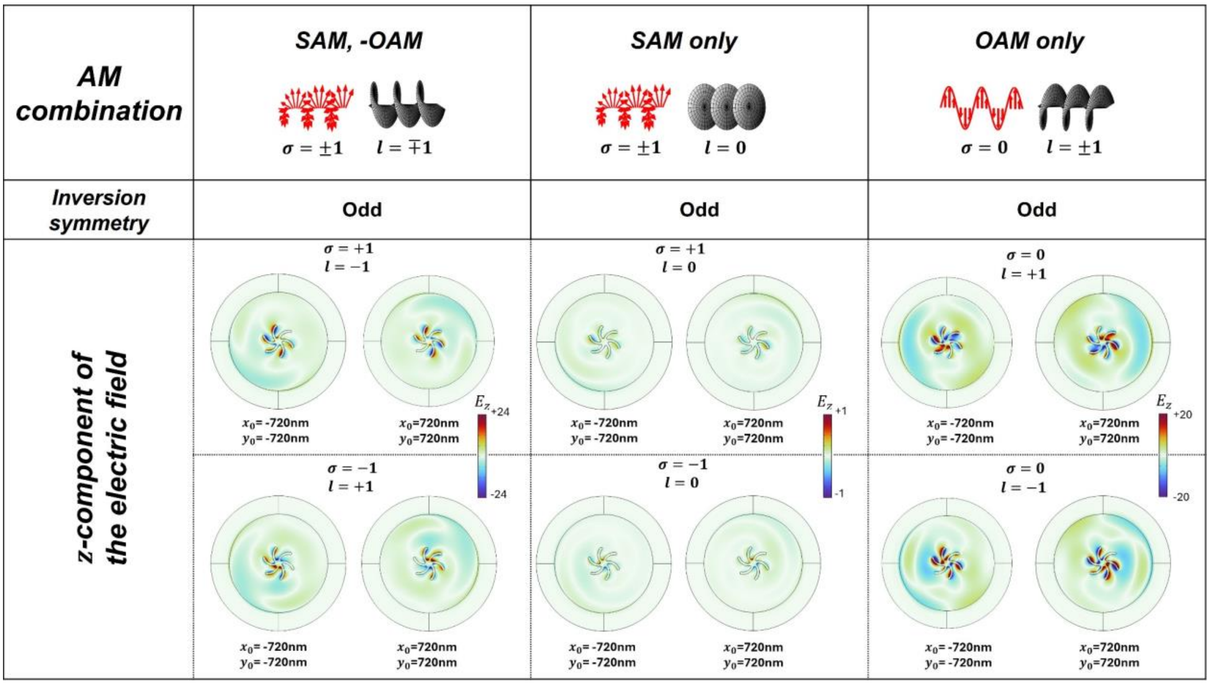

Figure S16: Simulated z-component of the electric field plotted when the weakly-focused beam is shifted by an x_0 and y_0 amounts in the x and y directions, respectively (positions 2 and 3 of Figure S14). The field is plotted on top of a RH shuriken structure for varying combinations of SAM and OAM ($\lambda = 590$ nm). The helicity parameter σ and topological charge l are indicated in the upper left corner of each panel.

References

1 Iketaki, Y., Watanabe, T., Bokor, N. & Fujii, M. Investigation of the center intensity of first- and second-order Laguerre-Gaussian beams with linear and circular polarization. *Opt. Lett.* **32**, 2357-2359 (2007). https://doi.org:10.1364/OL.32.002357

2 Forbes, K. A., Green, D. & Jones, G. A. Relevance of longitudinal fields of paraxial optical vortices. *Journal of Optics* **23** (2021).

3 Novotny, L. & Hecht, B. *Principles of Nano-Optics*. 2 edn, (Cambridge University Press, 2012).

4 Ganic, D., Gan, X. & Gu, M. Focusing of doughnut laser beams by a high numerical-aperture objective in free space. *Opt Express* **11**, 2747-2752 (2003). https://doi.org:10.1364/OE.11.002747

5 Gadegaard, N., Mosler, S. & Larsen, N. B. Biomimetic Polymer Nanostructures by Injection Molding. *Macromolecular Materials and Engineering* **288**, 76-83 (2003). https://doi.org:https://doi.org/10.1002/mame.200290037

6 Karimullah, A. S. *et al.* Disposable Plasmonics: Plastic Templated Plasmonic Metamaterials with Tunable Chirality. *Advanced Materials* **27**, 5610-5616 (2015). https://doi.org:https://doi.org/10.1002/adma.201501816

7 Hashiyada, S. & Tanaka, Y. Y. Rapid modulation of left- and right-handed optical vortices for precise measurements of helical dichroism. *Review of Scientific Instruments* **95**, 053101 (2024). https://doi.org:10.1063/5.0203715

8 Lalaguna, P. L. *et al.* Spatial Control of 2D Nanomaterial Electronic Properties Using Chiral Light Beams. *ACS Nano* **18**, 20401-20411 (2024). https://doi.org:10.1021/acsnano.4c04506

9 Rakić, A. D., Djurišić, A. B., Elazar, J. M. & Majewski, M. L. Optical properties of metallic films for vertical-cavity optoelectronic devices. *Appl Optics* **37**, 5271-5283 (1998). https://doi.org:10.1364/AO.37.005271

10 Sultanova, N., Kasarova, S. & Nikolov, I. Dispersion Properties of Optical Polymers. *ACTA PHYSICA POLONICA A* **116**, 585-587 (2009).

11 Richards, B. & Wolf, E. Electromagnetic diffraction in optical systems, II. Structure of the image field in an aplanatic system. *Proc. R. Soc. Lond.* **253**, 358-379 (1959). https://doi.org:10.1098/rspa.1959.0200

12 Wolf, E. & Gabor, D. Electromagnetic diffraction in optical systems - I. An integral representation of the image field. *Proceedings of the Royal Society of London. Series A. Mathematical and Physical Sciences* **253**, 349-357 (1959).
